# Extending the Glucosyl Ceramide Cassette Approach: Application in the Total Synthesis of Ganglioside GalNAc-GM1b

**DOI:** 10.3390/molecules181215153

**Published:** 2013-12-10

**Authors:** Miku Konishi, Akihiro Imamura, Kohki Fujikawa, Hiromune Ando, Hideharu Ishida, Makoto Kiso

**Affiliations:** 1 Department of Applied Bio-Organic Chemistry, Faculty of Applied Biological Sciences, Gifu University, 1-1 Yanagido, Gifu-shi, Gifu 501-1193, Japan; E-Mails: konishi@gifu-u.ac.jp (M.K.); kouki.fujikawa@riken.jp (K.F.); hando@gifu-u.ac.jp (H.A.); kiso@gifu-u.ac.jp (M.K.); 2 Institute for Integrated Cell-Material Sciences, Kyoto University, 69 Konoe-cho, Yoshida, Sakyo-ku, Kyoto 606-8501, Japan

**Keywords:** ganglioside, GalNAc-GM1b, total synthesis, cassette approach

## Abstract

The development of a novel cyclic glucosyl ceramide cassette acceptor for efficient glycolipid syntheses was investigated. *p*-Methoxybenzyl (PMB) groups were selected as protecting groups at C2 and C3 of the glucose residue with the aim of improving the functionality of the cassette acceptor. The choice of the PMB group resulted in a loss of β-selectivity, which was corrected by using an appropriate tether to control the spatial arrangement and the nitrile solvent effect. To investigate the effect of linker structure on the β-selectivity of intramolecular glycosylation, several linkers for tethering the glucose and ceramide moiety were designed and prepared, namely, succinyl, glutaryl, dimethylmalonyl, and phthaloyl esters. The succinyl ester linker was the best for accessing the cassette form. The newly designed glucosyl ceramide cassette acceptor was then applied in the total synthesis of ganglioside GalNAc-GM1b.

## 1. Introduction

Gangliosides, which are glycosphingolipids that contain one or more sialic acid residues, are components of all animal cell membranes and participate in many biological events, such as cell–cell interaction, signal transduction, immunological reaction, and neuronal differentiation [1,2,3]. Found in high abundance in the nervous system, several neuronal gangliosides have been linked with neurological disorders including Alzheimer’s disease, Parkinson’s disease, and Huntington’s disease [4]. Moreover, autoimmune neuropathy such as Guillain–Barré syndrome arises from the production of anti-ganglioside antibodies [5,6]. The growing body of research regarding the physiological and pathological implications of gangliosides has given rise to immense interest, not only among biologists, but also among synthetic chemists. Many synthetic organic chemists have contributed to developing methodology for the total synthesis of natural gangliosides and encountered several notable synthetic challenges, including regio- and stereo-selective sialylation and the introduction of the ceramide moiety into the oligosaccharide chain. Reliable methods for α-sialylation have been developed and used in numerous syntheses of natural gangliosides and analogues [7,8,9]; however, linking the flexible ceramide moiety to a large glycan remains a challenging undertaking. The typical procedure for connecting the lipid and glycan units is first to prepare the entire oligosaccharide framework and then to link it either to 2-azide sphingosine, which serves as a ceramide precursor, or to the ceramide moiety directly. This general procedure has proved effective for small gangliosides such as GM4 and GM3 [10,11]. In the synthesis of complex gangliosides, however, the oligosaccharide donor generally couples to the lipid acceptor in low yields. Our group has recently developed the glucosyl ceramide (GlcCer) cassette approach in order to overcome these synthetic challenges; our procedure involves coupling of glucose and ceramide (forming GlcCer) early in the total synthesis. This methodology has been used for efficiently synthesizing a series of natural gangliosides including GQ1b [12], GM3 [13], GalNAc-GD1a [14], X2 [15], and LLG-3 [16] in satisfactory overall yields. Having established a robust method for synthesizing gangliosides, we have shifted our attention to efficiently preparing GlcCer cassette acceptors. Two types of GlcCer cassette acceptors have been developed to date: an acyclic type [12,15,16] and a cyclic type [13,14]. Of these two types, acyclic cassettes are more reactive, but cyclic cassettes are easier to prepare. Against this background, we set out to develop a highly reactive cyclic GlcCer cassette acceptor. Here we describe the development of a novel cyclic GlcCer cassette acceptor and its application in the total synthesis of ganglioside GalNAc-GM1b.

## 2. Results and Discussion

### 2.1. Synthesis of a Novel Cyclic GlcCer Cassette Acceptor

#### 2.1.1. Design of a Novel Cyclic GlcCer Cassette Acceptor

**Figure 1:**
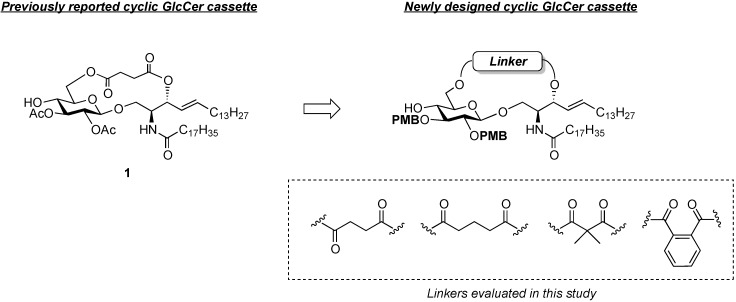
Structure of our previously reported cyclic GlcCer cassette acceptor (**left**). Structure of newly designed cyclic GlcCer cassette acceptor (**right**).

The structure of the previously used cyclic GlcCer (**1**) is shown in Figure 1. We speculated that the low reactivity of the 4-OH group of the glucose residue was due to the presence of the electron-withdrawing acetyl group at the C3 position. Thus, installing an electron-donating protecting group at the neighboring C3 position was expected to enhance the nucleophilicity of 4-OH. Furthermore, to retain a route for accessing the cassette, the same protecting groups should be installed on O2 and O3 of the glucose. Based on the above considerations, the *p*-methoxybenzyl (PMB) group was chosen as a protecting group because it can serve as an electron-donating group and be selectively removed under mild acidic conditions. A point of concern, however, was that the non-participating PMB group at the C2 position would cause a loss of stereoselectivity for the β-product in the intramolecular glycosylation. Therefore, we envisioned controlling stereoselectivity by means of a tethered structure; in particular, we anticipated that nucleophilic attack by the primary alcohol of the ceramide on the anomeric center of the glucose could be restricted to a single face and that the β-anomer could be selectively prepared. The four linkers evaluated in this study are shown in Figure 1: (1) succinyl ester, which is used in **1**; (2) glutaryl ester, which has a more flexible longer chain; (3) dimethylmalonyl ester, which has a bulkier shorter chain; and (4) phthaloyl ester, which has a more rigid chain.

#### 2.1.2. Preparation of Cyclic GlcCer Cassette Acceptors with Various Linkers

**Scheme 1:**
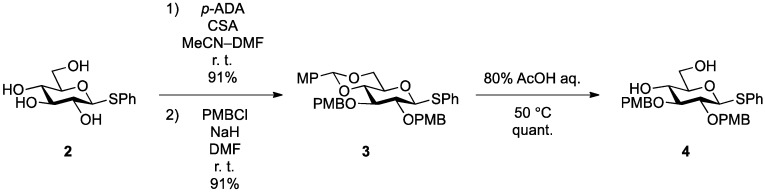
Synthesis of the 2,3-di-*O*-PMB-protected glucose derivative.

**Scheme 2:**
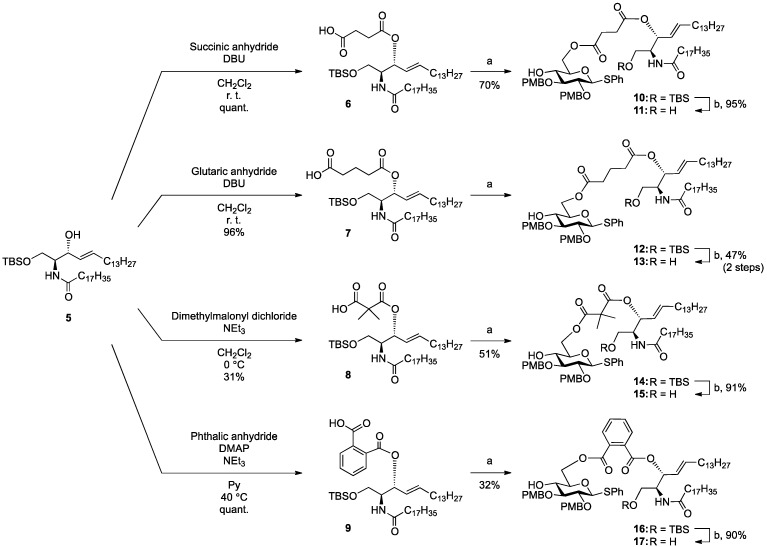
Tethering between the glucose residue and ceramide derivative by various types of dicarboxylate linkers.

As shown in Scheme 1, 2,3-di-*O*-*p*-methoxybenzyl-protected glucose derivative **4** was efficiently prepared from phenylthio-β-d-glucopyranoside **2** in three steps. 

Installation of anisylidene protecting groups at the C4 and C6 positions of **2** followed by introduction of PMB protecting groups at the C2 and C3 positions afforded fully protected glucose derivative **3** in excellent yield. Hydrolysis of the anisylidene group under acidic conditions gave diol **4**, which was ready for linking to the ceramide moiety.

Scheme 2 shows the procedure for linking glucose derivative **4** to the ceramide moiety. The 3-OH ceramide derivative **5** [13] was treated with succinic anhydride, glutaric anhydride, dimethylmalonyl chloride, or phthalic anhydride under optimized conditions to form the corresponding carboxylic acid derivatives **6**, **7**, **8**, or **9** in almost quantitative yield, except for compound **8**. 

*Reagents and conditions:* (a) **4**, EDC·HCl, DMAP, CH_2_Cl_2_, 0 °C → r.t.; (b) TBAF, AcOH, THF, 0 °C → r.t.

The preparation of **8** was hampered by an undesired main reaction that formed an isobutyrate product via decarboxylation. Succinic acid derivative **6** [13] was linked to glucose **4** in the presence of EDC·HCl and DMAP in CH_2_Cl_2_ at 0 °C, giving tethered product **10** in 70% yield. The *tert*-butyldimethylsilyl (TBS) group on **10** was removed by TBAF treatment to afford **11** in 95% yield. By the same procedure, compound **7** was linked to **4** to give **12**, along with a by-product in which the ceramide moiety was tethered to C4 of the glucose. Since these regioisomers were difficult to separate by column chromatography, the mixture was directly subjected to the next reaction without isolating the products. Upon removal of the TBS group, **13** was obtained in 47% yield over the two operations. Next, we attempted to form the dimethylmalonyl diester. After several attempts, we found that hetero-diester of dimethylmalonic acid was difficult to form and the best yield of the coupled product **14** was moderate (51%). Conversion of **14** into **15** proceeded smoothly in excellent yield. Lastly, phthalic acid derivative **9** was reacted with glucose **4** under the same conditions, providing the desired diester **16** in poor yield (32%). In this reaction, an unexpected phthalate product formed in which the C6 and C4 positions of the glucose residue were tethered. Subsequent removal of the TBS group furnished **17** in 90% yield.

#### 2.1.3. Intramolecular Glycosylation towards Novel Cyclic GlcCer Cassette Acceptors

The alcohols prepared in Scheme 2 were subjected to the intramolecular glycosylation to evaluate the β-selectivity of the reaction (Table 1). Intramolecular glycosylation of **11**, which contained the succinyl linker, was promoted by dimethyl(methylthio)sulfonium triflate [17,18] in CH_2_Cl_2_ at 0 °C. The reaction proceeded smoothly and afforded intramolecularly glycosylated **18** in 67% yield with poor anomeric selectivity (α/β = 1:1.7, entry 1). 

**Table 1:** Investigation into the effect of various linkers on intramolecular glycosylation.

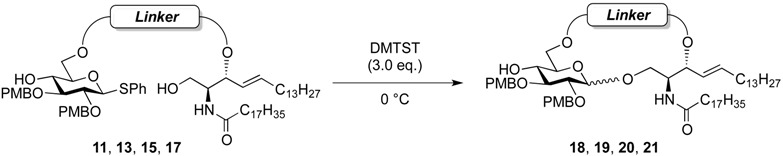						
Entry	Compd.	Linker	Condition *^a^*	Product	% Yield *^b^*	α/β Ratio
1	**11**	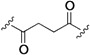	A	**18**	67	1/1.7
2	**11**		B	**18**	77	1/8.2
3	**13**	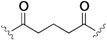	A	**19**	34	1/2.0
4	**13**		B	**19**	40	1/7.7
5	**15**	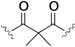	A	**20**	75	1/2.4
6	**15**		B	**20**	76	1/9.1
7	**17**	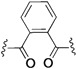	A	**21**	53	1/2.0
8	**17**		B	**21**	71	1/5.2

*^a^ Condition A***:** CH_2_Cl_2_, molecular sieve 4 Å; *Condition B***:** CH_3_CN–CH_2_Cl_2_ (2:1), molecular sieve 3 Å; *^b^* Isolated yield. DMTST: dimethyl(methylthio)sulfonium trifluoromethanesulfonate.

Acetonitrile, which generally promotes β-selective glycosylation, was examined as the main solvent: as expected, the nitrile solvent effect gave improved β-selectivity (α/β = 1:8.2, entry 2). Note that the desired β-product could be purified by recrystallization in the case of **18** only. Similarly, intramolecular glycosylation of glutaryl ester-tethered **13** was performed (entries 3 and 4). The longer more flexible linker compared with the one in **11** reduced both the yield and the stereoselectivity of the glycosylation (77%, α/β = 1:8.2 *vs*. 40%, α/β = 1:7.7). Compound **15**, which had the shortest linker, was used to examine whether the chain length of the linker would affect the stereochemical outcome of the intramolecular glycosylation. The β-selectivity for intramolecular glycosylation of **15** was only slightly better than that of **11**. Also, the α- and β-anomers were difficult to separate. Considering that a flexible linker appeared to hinder β-selectivity, we turned our attention to the compound with a more rigid linker, namely, compound **17**, which contained a rigid phthaloyl linker that could suppress free rotation around the tether (entries 7 and 8). As a result, a slight shift toward α-selectivity was observed (entry 8, α/β = 1:5.2).

Contemplating the above results, we considered that the succinyl linker might be the best for accessing the desired novel cyclic GlcCer cassette acceptor. Next, the novel GlcCer cassette acceptor **18β** was utilized for the total synthesis of ganglioside GalNAc-GM1b to investigate its applicability to glycolipid synthesis.

### 2.2. Total Synthesis of Ganglioisde GalNAc-GM1b

#### 2.2.1. Assembly of the Non-Reducing End Pentasaccharide Donor

**Figure 2:**
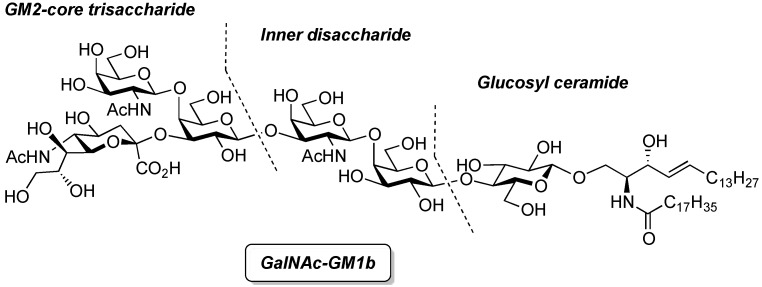
Structure of ganglioside GalNAc-GM1b and key disconnections for total synthesis.

Ganglioside GalNAc-GM1b was first isolated from Tay–Sachs brain in 1981 [19] and from murine T lymphocytes in 1989 [20], and has been suggested to play important roles in the mammalian immune system. Furthermore, immunoglobulin M monoclonal antibody against GalNAc-GM1b has been isolated from patients with Guillain–Barré syndrome [21,22,23]. Having been implicated in these intractable diseases, GalNAc-GM1b has elicited much interest. The chemical total synthesis of GalNAc-GM1b was achieved first by Ogawa and co-workers in 1990 [24], who adopted the standard procedure for introducing the ceramide moiety into the glycan. Although their construction of the glycan sequence was elegant, the final coupling of the ceramide acceptor and hexasaccharide donor was accomplished in with a low yield of 15%. Thus, we decided to apply our novel cyclic GlcCer cassette to the total synthesis of GalNAc-GM1b in order to extend the generality of the GlcCer cassette approach (Figure 2).

**Scheme 3:**
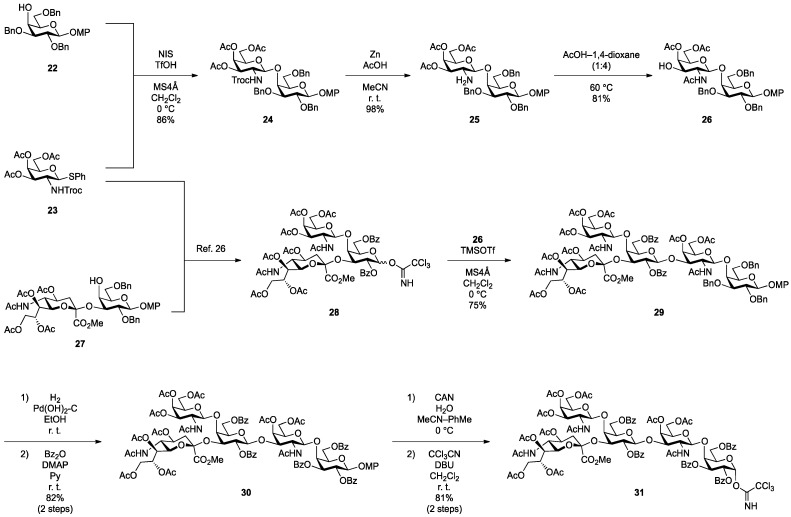
Synthesis of the non-reducing end glycan sequence of GalNAc-GM1b.

The non-reducing end glycan sequence of GalNAc-GM1b was efficiently prepared as shown in Scheme 3. Glycosylation of known galactosyl acceptor **22** [25] with galactosaminyl donor **23** [26] was carried out in the presence of NIS and TfOH [27,28] in CH_2_Cl_2_ at 0 °C, giving disaccharide **24** in 86% yield. Reductive removal of the Troc group by treatment with zinc gave **25** in excellent yield. Then, under optimized acidic conditions (AcOH/1,4-dioxane: 1:4; 60 °C), acetyl migration from the C3 position of the galactosamine residue to the liberated amine was achieved in good yield (inner disaccharide acceptor **26**, 81%) [14]. For efficient migration of the acetyl group, the selected solvent and AcOH/solvent ratio were important. Also, undesired migration of an acetyl group from C4 to C3 of the galactosamine was observed, giving in 8% yield a disaccharide with an unprotected 4-OH group in the galactosamine residue. The GM2-core trisaccharide donor **28** was prepared from **23** and the sialylα(2,3)galactose unit **27** according to a previously reported procedure [26]. The coupling of **28** and **26** was promoted by a catalytic amount of TMSOTf in CH_2_Cl_2_ at 0 °C to give pentasaccharide **29** in 75% yield. The benzyl groups in **29** were replaced with benzoyl groups by hydrogenation and subsequent benzoylation, affording **30** in good yield. Selective removal of the *p*-methoxyphenyl (MP) group with CAN [29] followed by introduction of the trichloroacetimidate leaving group [30] gave the non-reducing end glycan donor **31** in 91% yield over two steps.

#### 2.2.2. Final Glycosylation by the GlcCer Cassette Approach and Global Deprotection

**Scheme 4:**
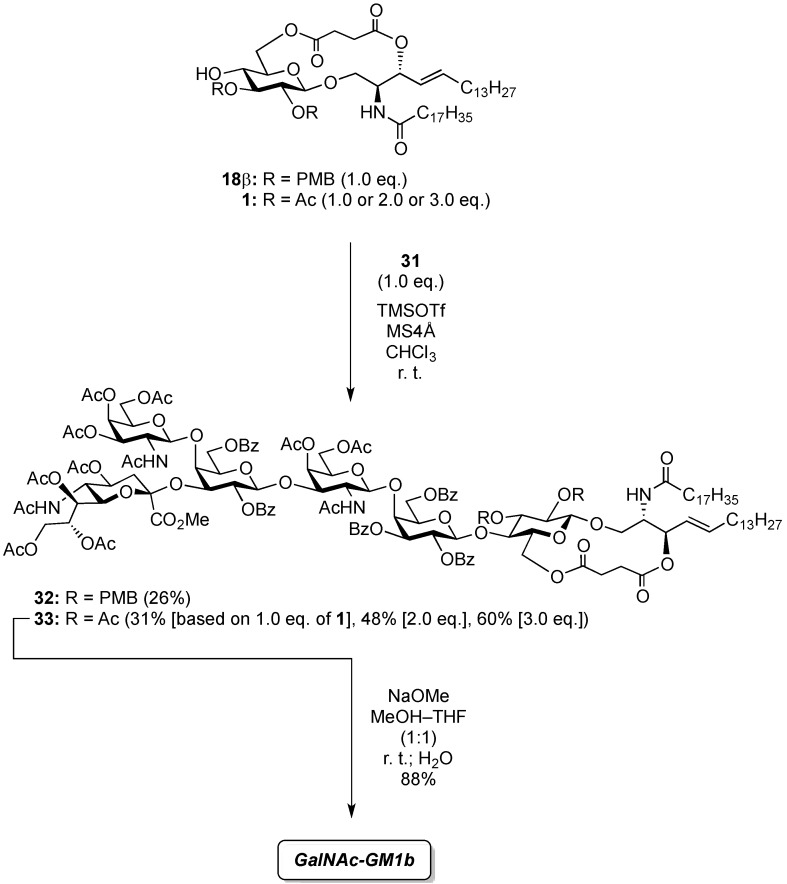
Final glycosylation using the GlcCer cassette approach and global deprotection.

First, the novel cyclic GlcCer cassette acceptor **18β** was glycosylated with **31** in the presence of TMSOTf in CHCl_3_ at room temperature, giving the fully protected GalNAc-GM1b framework in a meager 26% yield. In this glycosylation, most of the donor was hydrolyzed to form the corresponding hemiacetal compound and *ca.* 67% of the acceptor were recovered. Contrary to our expectations, the acceptor equipped with the electron-donating PMB groups at the C2 and C3 positions of the glucose residue did not serve as a good cassette. Although we cannot explain this low yield with certainty, we speculate that the functionality at C2 might significantly lower the nucleophilicity of the 4-OH group because similar 2-*O*-benzoyl-protected cyclic GlcCer acceptor, which only differed by the protecting group at O-2 compared to **18β**, served as a good acceptor in our previous experiment [14]. Next, the previously reported GlcCer cassette **1** [14] was used as an alternative for the final glycosylation. When 1.0 eq. of **1** was used as the glycosyl acceptor, the desired GalNAc-GM1b framework (**33**) was obtained in 31% yield. Increasing the equivalent amount of the acceptor increased the coupling yield to 60%. Finally, global deprotection to remove the acetyl, benzoyl, and succinyl groups was performed by treatment with NaOMe in MeOH/THF (1:1) followed by addition of water, furnishing the target ganglioside GalNAc-GM1b in 88% yield (Scheme 4).

## 3. Experimental

### General Methods

All reactions were carried out under a positive pressure of argon, unless otherwise noted. All chemicals were purchased from commercial suppliers and used without further purification, unless otherwise noted. Molecular sieves were purchased from Wako Chemicals Inc. (Miyazaki, Japan) and dried at 300 °C for 2 h in a muffle furnace prior to use. Solvents as reaction media were dried over molecular sieves and used without purification. TLC analysis was performed on Merck TLC plates (silica gel 60F254 on glass plate). Compound detection was either by exposure to UV light (2536 Å) or by soak in a solution of 10% H_2_SO_4_ in ethanol followed by heating. Silica gel (80 mesh and 300 mesh) manufactured by Fuji Silysia Co. was used for flash column chromatography. Quantity of silica gel was usually estimated as 200 to 400-fold weight of sample to be charged. Solvent systems in chromatography were specified in *v*/*v*. Evaporation and concentration were carried out *in vacuo*. ^1^H-NMR and ^13^C-NMR spectra were recorded with JEOL ECA 400/500/600 and Bruker UltraShield Plus 500 spectrometers. Chemical shifts in ^1^H-NMR spectra are expressed in ppm (δ) relative to the signal of Me_4_Si, adjusted to δ 0.00 ppm. Data are presented as follow: Chemical shift, multiplicity (s = singlet, d = doublet, t = triplet, dd = double of doublet, td = triple doublet, m = multiplet and/or multiple resonances), integration, coupling constant in Hertz (Hz), position of the corresponding proton. COSY methods were used to confirm the NMR peak assignments. High-resolution mass (ESI-TOF MS) spectra were run in a Bruker micrOTOF. Optical rotations were measured with a “Horiba SEPA-300” high-sensitive polarimeter.


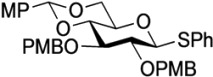


*Phenyl 4,6-O-anisylidene-2,3-di-O-p-methoxybenzyl-1-thio-β-d-glucopyranoside* (**3**). To the solution of **2** (20.0 g, 73.5 mmol) in the mixed solvent (CH_3_CN–DMF 735 mL:200 mL) were added *p*-anisaldehyde dimethyl acetal (25.0 mL, 147 mmol) and (±)-camphor-10-sulfonic acid (CSA) (680 mg, 2.94 mmol) at 0 °C. After stirring for 2.5 h at room temperature as the reaction was monitored by TLC (10:1 CHCl_3_–MeOH), the reaction was quenched by the addition of triethylamine. The reaction mixture was concentrated and diluted with EtOAc, of which solution was then added to separatory funnel. After addition of distilled water to the solution, the desired 4,6-*O*-anisylidenated product was appeared as a pure crystalline material (26.0 g, 91%), the physical data of which was identical to those reported in the literature [31]. To a solution of the 4,6-*O*-anisylidenated product obtained (2.00 g, 5.13 mmol) in DMF (25.7 mL) was added sodium hydride (492 mg, 20.5 mmol) at 0 °C. After stirring for 1 h at 0 °C, *p*-methoxybenzyl chloride (2.8 mL, 20.5 mmol) was added to the mixture. After stirring for 17 h at rt as the reaction was monitored by TLC (1:2.5 EtOAc–*n*-hexane), the reaction was quenched by MeOH at 0 °C. Dilution of the mixture with EtOAc provided a solution, which was then washed with H_2_O, satd aq NaHCO_3_ and brine. The organic layer was subsequently dried over Na_2_SO_4_ and concentrated. The resulting residue was purified by silica gel column chromatography (1:3 EtOAc–*n*-hexane) to give **3** (2.94 g, 91%). [α]_D_ +3.3° (c 0.3, CHCl_3_); ^1^H-NMR (500 MHz, CDCl_3_) δ 7.54–6.82 (m, 17H, 4Ar), 5.54 (s, 1H, ArC*H*), 4.85 (d, 1H, *J*_gem_ = 10.9 Hz, ArC*H*_2_), 4.78–4.70 (m, 4H, ArC*H*_2_, H-1), 4.35 (dd, 1H, *J*_5,6_ = 5.2 Hz, *J*_gem_ = 10.3 Hz, H-6), 3.81–3.76 (m, 11H, 3OCH_3_, H-3, H-6'), 3.66 (t, 1H, *J*_3,4_ = *J*_4,5_ = 9.5 Hz, H-4), 3.45 (m, 2H, H-2, H-5); ^13^C-NMR (125 MHz, CDCl_3_) δ 160.0, 159.4, 159.3, 133.1, 132.2, 130.5, 130.2, 129.9, 129.8, 129.0, 127.8, 127.3, 113.8, 113.6, 101.1, 88.3, 82.7, 81.4, 80.1, 75.5, 74.9, 70.2, 68.6, 55.3, 55.2. HRMS (ESI) *m*/*z*: found [M+Na]^+^ 653.2180, C_36_H_38_O_8_S calcd for [M+Na]^+^ 653.2180.


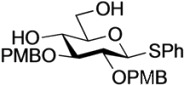


*Phenyl 2,3-di-O-p-methoxybenzyl-1-thio-β-d-glucopyranoside* (**4**). Compound **3** (2.60 g, 4.13 mmol) was dissolved in 80% AcOH aq (41.3 mL) and the solution was stirred for 1.5 h at 50 °C as the reaction was monitored by TLC (2:1 EtOAc–*n*-hexane). The reaction mixture was diluted with EtOAc and carefully washed with ice-cooled satd aq Na_2_CO_3_ and brine. The organic layer was subsequently dried over Na_2_SO_4_ and concentrated. The resulting residue was purified by silica gel column chromatography (1:1 EtOAc–*n*-hexane) to give **4** (2.11 g, quant.). [α]_D_ −18.0° (c 0.4, CHCl_3_); ^1^H-NMR (500 MHz, CDCl_3_) δ 7.52–6.87 (m, 13H, 3Ar), 4.90 (d, 1H, *J*_gem_ = 11.3 Hz, ArC*H*_2_), 4.88 (d, 1H, *J*_gem_ = 11.0 Hz, ArC*H*_2_), 4.70 (m, 2H, ArC*H*_2_, H-1), 4.64 (d, 1H, ArC*H*_2_), 3.86 (m, 1H, H-6), 3.80 (2 s, 6H, 2OCH_3_), 3.73 (m, 1H, H-6'), 3.55–3.43 (m, 3H, H-4, H-3, H-2), 3.32 (m, 1H, H-5), 2.28 (d, 1H, *J*_4,OH_ = 2.5 Hz, OH), 2.08 (t, 1H, *J*_6,OH_ = *J*_6',OH_ = 6.6 Hz, OH); ^13^C-NMR (125 MHz, CDCl_3_) δ 159.5, 159.5, 133.6, 131.7, 130.4, 130.0, 129.9, 129.7, 129.6, 129.0, 127.6, 114.1, 113.9, 87.7, 85.6, 80.6, 79.1, 75.0, 75.0, 70.4, 62.8, 55.3, 55.3. HRMS (ESI) *m*/*z*: found [M+Na]^+^ 535.1758, C_28_H_32_O_7_S calcd for [M+Na]^+^ 535.1761.


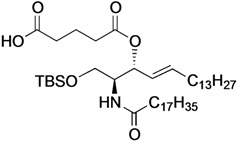


*(2S,3R,4E)-1-O-tert-Butyldimethylsilyl-3-O-(4-hydroxycarbonylbutanoate)-2-octadecanamido-4-octa-decene-1,3-diol* (**7**). To a solution of **5** (25 mg, 36.8 µmol) in CH_2_Cl_2_ (368 µL) were added glutaric anhydride (21 mg, 184 µmol) and DBU (11 µL, 73.6 µmol) at 0 °C. After stirring for 1 h at rt as the reaction was monitored by TLC (4:1 diethylether–*n*-hexane), the reaction was quenched by the addition of MeOH at 0 °C. The reaction mixture was evaporated. The crude residue obtained was purified by silica gel column chromatography (1:3 diethylether–*n*-hexane) to give **7** (28 mg, 96%). [α]_D_ +4.7° (c 0.3, CHCl_3_); ^1^H-NMR (500 MHz, CDCl_3_) δ 5.76 (m, 2H, H-5, NH), 5.42 (dd, 1H, *J*_3,4_ = 7.2 Hz, *J*_4,5_ = 15.3 Hz, H-4), 5.32 (t, 1H, *J*_2,3_ = 7.2 Hz, H-3), 4.28 (m, 1H, H-2), 3.72 (dd, 1H, *J*_1,2_ = 3.1 Hz, *J*_gem_ = 10.3 Hz, H-1), 3.59 (dd, 1H, *J*_1',2_ = 4.4 Hz, H-1'), 2.46–2.36 (m, 4H, 2C(=O)CH_2_), 2.17 (m, 2H, C(=O)CH_2_*^Cer^*), 2.01 (m, 2H, H-6, H-6'), 1.95 (m, 2H, -CH_2_-), 1.59 (m, 2H, C(=O)CH_2_C*H_2_*), 1.25 (m, 50H, 25-CH_2_-*^Cer^*), 0.88 (m, 15H, *t*-Bu, 2-CH_3_*^Cer^*), 0.05-0.04 (2 s, 6H, Si(CH_3_)_2_); ^13^C-NMR (125 MHz, CDCl_3_) δ 177.3, 173.3, 173.1, 171.1, 136.9, 124.6, 73.7, 61.6, 51.8, 51.6, 37.0, 33.4, 33.1, 33.0, 32.9, 32.4, 31.9, 30.0, 29.7, 29.7, 29.5, 29.5, 29.4, 29.4, 29.3, 29.2, 29.0, 25.8, 25.7, 22.7, 20.1, 19.9, 18.2, 14.1, −5.6, −5.6. HRMS (ESI) *m*/*z*: found [M+Na]^+^ 816.6507, C_47_H_91_NO_6_Si calcd for [M+Na]^+^ 816.6508.


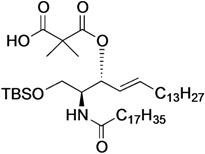


*(2S,3R,4E)-1-O-tert-Butyldimethylsilyl-3-O-(2-hydroxycarbonylisobutanoate)-2-octadecanamido-4-octadecene-1,3-diol* (**8**)*.* To a solution of **5** (47 mg, 69.2 µmol) in CH_2_Cl_2_ (1.4 mL) were added dimethylmalonyl dichloride (92 µL, 692 µmol) and triethylamine (96 µL, 692 µmol) at 0 °C. After stirring for 3 h at 0 °C as the reaction was monitored by TLC (1:1 diethylether–*n*-hexane), the reaction was diluted with CHCl_3_. The solution was then washed with H_2_O and brine. The organic layer was subsequently dried over Na_2_SO_4_, and concentrated. The resulting residue was purified by silica gel column chromatography (1:4 diethylether–*n*-hexane) to give **8** (17 mg, 31%). [α]_D_ +4.4° (c 0.8, CHCl_3_); ^1^H-NMR (500 MHz, CDCl_3_) δ 6.44 (d, 1H, *J*_2,NH_ = 9.0 Hz, NH), 5.77 (m, 1H, H-5), 5.41 (dd, 1H, *J*_3,4_ = 7.4 Hz, *J*_4,5_ = 15.3 Hz, H-4), 5.32 (t, 1H, *J*_2,3_ = 7.4 Hz, H-3), 4.20 (m, 1H, H-2), 3.76 (dd, 1H, *J*_1,2_ = 2.7 Hz, *J*_gem_ = 10.3 Hz, H-1), 3.62 (dd, 1H, *J*_1',2_ = 4.2 Hz, H-1'), 2.28 (m, 2H, C(=O)CH_2_), 2.00 (m, 2H, H-6, H-6'), 1.60 (m, 2H, C(=O)CH_2_C*H_2_*), 1.49 (s, 6H, CH_3_CCH_3_), 1.25 (m, 50H, 25-CH_2_-), 0.89 (m, 15H, *t*-Bu, 2-CH_3_*^Cer^*), 0.05 (2 s, 6H, Si(CH_3_)_2_). LRMS (ESI) *m*/*z*: found [M−H]^−^ 792.6438, C_47_H_91_NO_6_Si calcd for [M−H]^−^ 792.6543.


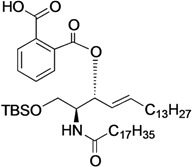


*(2S,3R,4E)-1-O-tert-Butyldimethylsilyl-3-O-(o-hydroxycarbonylbenzoate)-2-octadecanamido-4-octadecene-1,3-diol* (**9**). To a solution of **5** (1.00 g, 1.47 mmol) in pyridine (7.4 mL) were added phthalic anhydride (327 mg, 2.21 mmol), DMAP (18 mg, 147 µmol) and triethylamine (612 µL, 4.41 mmol) at 0 °C. After stirring for 23 h at 40 °C as the reaction was monitored by TLC (4:1 diethylether–*n*-hexane), the solvent was removed by co-evaporation with toluene, and then the residue was diluted with CHCl_3_, washed with H_2_O. The organic layer was subsequently dried over Na_2_SO_4_, and concentrated. The resulting residue was purified by silica gel column chromatography (1:2 diethylether–*n*-hexane) to give **9** (1.22 g, quant.). [α]_D_ +58.3° (c 0.3, CHCl_3_); ^1^H-NMR (600 MHz, CDCl_3_) δ 7.80–7.52 (m, 4H, Ar), 6.07 (d, 1H, *J*_2,NH_ = 10.3 Hz, NH), 5.81 (m, 1H, H-5), 5.71 (t, 1H, *J*_2,3_ = *J*_3,4_ = 6.9 Hz, H-3), 5.54 (dd, 1H, *J*_4,5_ = 15.5 Hz, H-4), 4.44 (m, 1H, H-2), 3.76 (dd, 1H, *J*_1,2_ = 4.1 Hz, *J*_gem_ = 10.3 Hz, H-1), 3.64 (dd, 1H, *J*_1',2_ = 5.5 Hz, H-1'), 2.27 (m, 2H, C(=O)CH_2_), 2.03 (m, 2H, H-6, H-6'), 1.63 (m, 2H, C(=O)CH_2_C*H_2_*), 1.33 (m, 50H, 25-CH_2_-), 0.88 (m, 15H, *t*-Bu, 2-CH_3_), 0.05 (2 s, 6H, Si(CH_3_)_2_); ^13^C-NMR (150 MHz, CDCl_3_) δ 174.7, 169.8, 166.4, 136.9, 132.7, 131.4, 131.2, 130.8, 129.5, 128.8, 123.7, 75.3, 62.1, 52.3, 37.0, 32.4, 31.9, 29.7, 29.7, 29.5, 29.4, 29.4, 29.3, 29.3, 29.0, 25.8, 25.7, 22.7, 18.1, 14.1, −5.5, −5.6. HRMS (ESI) *m*/*z*: found [M+Na]^+^ 850.6351, C_51_H_91_NO_5_Si calcd for [M+Na]^+^ 850.6351.


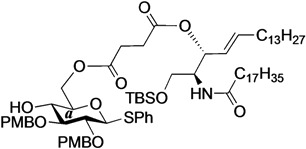


*Phenyl 6-O-{3-[(2S,3R,4E)-1-O-tert-butyldimethylsilyl-2-octadecanamido-4-octadecene-3-yloxy-1,3-diol]carbonylpropanoyl}-2,3-di-O-p-methoxybenzyl-1-thio-β-d-glucopyranoside* (**10**). To a solution of **4** (124 mg, 242 µmol) in CH_2_Cl_2_ (4.8 mL) were added **6** (200 mg, 242 µmol), EDC·HCl (51 mg, 266 µmol) and DMAP (3.0 mg, 24.2 µmol) at 0 °C. After stirring for 2.5 h at rt as the reaction was monitored by TLC (1:1 EtOAc–*n*-hexane), the mixture was diluted with CHCl_3_. The solution was then washed with H_2_O. The organic layer was subsequently dried over Na_2_SO_4_ and concentrated. The resulting residue was purified by silica gel column chromatography (1:5 EtOAc–*n*-hexane) to give **10** (215 mg, 70%). [α]_D_ −7.7° (c 0.3, CHCl_3_); ^1^H-NMR (500 MHz, CDCl_3_) δ 7.56–7.24 (m, 5H, Ph), 7.54–6.85 (m, 8H, 2Ar), 5.74 (m, 2H, H-5*^Cer^*, NH*^Cer^*), 5.43 (dd, 1H, *J*_3,4_ = 7.2 Hz, *J*_4,5_ = 15.2 Hz, H-4*^Cer^*), 5.36 (t, 1H, *J*_2,3_ = 7.2 Hz, H-3*^Cer^*), 4.84 (d, 1H, *J*_gem_ = 11.0 Hz, ArC*H*_2_), 4.83 (d, 1H, *J*_gem_ = 10.0 Hz, ArC*H*_2_), 4.77 (d, 1H, ArC*H*_2_), 4.68 (d, 1H, ArC*H*_2_), 4.65 (d, 1H, *J*_1,2_ = 9.2 Hz, H-1a), 4.39 (dd, 1H, *J*_5,6_ = 4.6 Hz, *J*_gem_ = 12.0 Hz, H-6a), 4.33 (dd, 1H, *J*_5,6'_ = 2.0 Hz, H-6'a), 4.23 (m, 1H, H-2*^Cer^*), 3.80–3.79 (2 s, 6H, 2 OCH_3_), 3.69 (dd, 1H, *J*_1,2_ = 9.7 Hz, *J*_gem_ = 10.4 Hz, H-1*^Cer^*), 3.57 (m, 2H, H-1'*^Cer^*, H-4a), 3.50 (t, 1H, *J*_2,3_ = *J*_3,4_ = 9.2 Hz, H-3a), 3.44 (m, 3H, H-2a, H-5a, OHa), 2.66–2.60 (m, 4H, 2C(=O)CH_2_), 2.15 (m, 2H, C(=O)CH_2_*^Cer^*), 2.01 (m, 2H, H-6*^Cer^*, H-6'*^Cer^*), 1.57 (m, 2H, C(=O)CH_2_C*H*_2_), 1.30 (m, 50H, 25-CH_2_-), 0.88 (m, 15H, *t*-Bu, 2-CH_3_*^Cer^*), 0.04 (2 s, 6H, Si(CH_3_)_2_); ^13^C-NMR (125 MHz, CDCl_3_) δ 172.8, 172.5, 170.7, 159.4, 159.3, 136.7, 133.7, 131.9, 130.6, 130.2, 129.9, 129.6, 128.8, 127.5, 124.6, 113.9, 113.8, 87.7, 85.6, 80.2, 77.5, 75.2, 75.0, 74.0, 69.8, 63.6, 61.6, 55.2, 55.2, 51.9, 36.9, 32.3, 31.9, 30.0, 29.6, 29.5, 29.5, 29.5, 29.4, 29.3, 29.2, 29.0, 29.0, 25.8, 25.8, 22.6, 18.1, 14.2, 14.1, −5.5, −5.6. HRMS (ESI) *m*/*z*: found [M+Na]^+^ 1296.8116, C_74_H_119_NO_12_SSi calcd for [M+Na]^+^ 1296.8114.


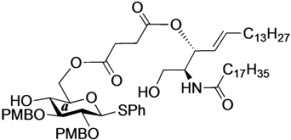


*Phenyl 6-O-{3-[(2S,3R,4E)-2-octadecanamido-4-octadecene-3-yloxy-1,3-diol]carbonylpropanoyl}-2,3-di-O-p-methoxybenzyl-1-thio-β-d-glucopyranoside* (**11**). To a solution of **10** (183 mg, 144 µmol) in THF (1.4 mL) were added AcOH (26 µL, 432 µmol) and TBAF (432 µL, 432 µmol) at 0 °C. After stirring for 2 h at rt as the reaction was monitored by TLC (1:1 EtOAc–*n*-hexane), the mixture was diluted with CHCl_3_. The solution was then washed with satd. aq. NaHCO_3_. The organic layer was subsequently dried over Na_2_SO_4_ and concentrated. The resulting residue was purified by silica gel column chromatography (1:1.5 EtOAc–*n*-hexane) to give **11** (159 mg, 95%). [α]_D_ −12.0° (c 0.3, CHCl_3_); ^1^H-NMR (500 MHz, CDCl_3_) δ 7.55–7.25 (m, 5H, Ph), 7.35–6.86 (m, 8H, 2Ar), 6.05 (d, 1H, *J*_2,NH_ = 9.0 Hz, NH*^Cer^*), 5.76 (m, 1H, H-5*^Cer^*), 5.42 (dd, 1H, *J*_3,4_ = 7.3 Hz, *J*_4,5_ = 15.2 Hz, H-4*^Cer^*), 5.35 (t, 1H, *J*_2,3_ = 7.3 Hz, H-3*^Cer^*), 4.85 (d, 1H, *J*_gem_ = 10.9 Hz, ArC*H*_2_), 4.84 (d, 1H, *J*_gem_ = 10.4 Hz, ArC*H*_2_), 4.72 (d, 1H, ArC*H*_2_), 4.68 (d, 1H, ArC*H*_2_), 4.66 (d, 1H, *J*_1,2_ = 9.6 Hz, H-1a), 4.45 (dd, 1H, *J*_5,6_ = 4.6 Hz, *J*_gem_ = 11.9 Hz, H-6a), 4.29 (dd, 1H, *J*_5,6'_ = 1.6 Hz, H-6'a), 4.15 (m, 1H, H-2*^Cer^*), 3.80–3.79 (2 s, 6H, 2OCH_3_), 3.66 (dd, 1H, *J*_1,2_ = 4.2 Hz, *J*_gem_ = 11.6 Hz, H-1*^Cer^*), 3.60 (near dd, 1H, H-1'*^Cer^*), 3.54–3.42 (m, 4H, H-3a, H-5a, H-4a, H-2a), 3.27 (br s, 1H, OHa), 2.83 (br s, 1H, OH*^Cer^*), 2.72–2.62 (m, 4H, 2C(=O)CH_2_), 2.16 (m, 2H, C(=O)CH_2_*^Cer^*), 2.11 (m, 2H, H-6*^Cer^*, H-6'*^Cer^*), 1.58 (m, 2H, C(=O)CH_2_C*H*_2_), 1.30 (m, 50H, 25-CH_2_-), 0.88 (m, 6H, 2-CH_3_*^Cer^*); ^13^C-NMR (125 MHz, CDCl_3_) δ 173.6, 172.7, 171.5, 159.4, 137.2, 133.7, 131.8, 130.4, 130.1, 129.9, 129.7, 128.9, 127.5, 124.5, 114.0, 113.8, 87.8, 85.6, 80.2, 77.3, 75.3, 75.0, 74.6, 69.6, 63.7, 61.6, 55.3, 55.2, 52.9, 36.7, 32.3, 31.9, 29.7, 29.6, 29.6, 29.5, 29.5, 29.4, 29.3, 29.3, 29.2, 29.1, 28.9, 25.7, 22.7, 14.1. HRMS (ESI) *m*/*z*: found [M+Na]^+^ 1182.7250, C_68_H_105_NO_12_S calcd for [M+Na]^+^ 1182.7250.


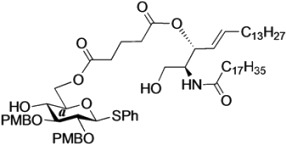


*Phenyl 6-O-{4-[(2S,3R,4E)-2-octadecanamido-4-octadecene-3-yloxy-1,3-diol]carbonylbutanoyl}-2,3-di-O-p-methoxybenzyl-1-thio-β-d-glucopyranoside* (**12**). To a solution of **4** (90 mg, 175 µmol) in CH_2_Cl_2_ (3.5 mL) were added **7** (139 mg, 175 µmol), EDC·HCl (37 mg, 193 µmol) and DMAP (2.1 mg, 17.5 µmol) at 0 °C. After stirring for 1.5 h at rt as the reaction was monitored by TLC (1:1 EtOAc–*n*-hexane), the mixture was diluted with CHCl_3_. The solution was then washed with H_2_O. The organic layer was subsequently dried over Na_2_SO_4_ and concentrated. The resulting residue was purified by silica gel column chromatography (1:5 EtOAc–*n*-hexane) to give the crude mixture mainly containing **12**. The crude mixture was exposed to high vacuum for 15 h and then dissolved in THF (1.8 mL). AcOH (31 µL, 525 µmol) and TBAF (525 µL, 525 µmol) were added to the mixture at 0 °C. After stirring for 4 h at rt as the reaction was monitored by TLC (1:1 EtOAc–*n*-hexane), the mixture was diluted with CHCl_3_. The solution was then washed with satd aq NaHCO_3_. The organic layer was subsequently dried over Na_2_SO_4_ and concentrated. The resulting residue was purified by silica gel column chromatography (1:2 EtOAc–*n*-hexane) to give **13** (96 mg, 47% in two steps). [α]_D_ −22.5° (c 1.0, CHCl_3_); ^1^H-NMR (500 MHz, CDCl_3_) δ 7.55–7.25 (m, 5H, Ph), 7.36–6.87 (m, 8H, 2Ar), 6.00 (d, 1H, *J*_2,NH_ = 8.6 Hz, NH*^Cer^*), 5.76 (m, 1H, H-5*^Cer^*), 5.44 (dd, 1H, *J*_3,4_ = 7.4 Hz, *J*_4,5_ = 15.4 Hz, H-4*^Cer^*), 5.28 (t, 1H, *J*_2,3_ = 7.4 Hz, H-3*^Cer^*), 4.87 (d, 1H, *J*_gem_ = 11.1 Hz, ArC*H*_2_), 4.85 (d, 1H, *J*_gem_ = 9.9 Hz, ArC*H*_2_), 4.69 (d, 1H, ArC*H*_2_), 4.68 (d, 1H, ArC*H*_2_), 4.65 (d, 1H, *J*_1,2_ = 9.4 Hz, H-1a), 4.34 (near s, 2H, H-6a, H-6'a), 4.12 (m, 1H, H-2*^Cer^*), 3.81–3.80 (2 s, 6H, 2OCH_3_), 3.66 (dd, 1H, *J*_1,2_ = 3.8 Hz, *J*_gem_ = 11.7 Hz, H-1*^Cer^*), 3.60 (dd, 1H, *J*_1',2_ = 2.7 Hz, H-1'*^Cer^*), 3.50–3.42 (m, 4H, H-2a, H-3a, H-4a, H-5a), 2.85 (br s, 1H, OH*^Cer^*), 2.75 (s, 1H, OHa), 2.43–2.39 (m, 4H, 2C(=O)CH_2_), 2.16 (m, 2H, C(=O)CH_2_*^Cer^*), 2.01 (m, 2H, H-6*^Cer^*, H-6'*^Cer^*), 1.96 (m, 2H, -CH_2_-), 1.58 (m, 2H, C(=O)CH_2_C*H*_2_), 1.29 (m, 50H, 25-CH_2_-*^Cer^*), 0.88 (m, 6H, 2-CH_3_*^Cer^*); ^13^C-NMR (125 MHz, CDCl_3_) δ 173.4, 173.1, 172.7, 159.5, 159.4, 137.4, 133.7, 131.9, 130.4, 130.0, 129.9, 129.7, 128.9, 127.6, 124.7, 114.1, 113.9, 87.7, 85.5, 80.3, 77.6, 75.2, 75.0, 74.3, 69.9, 63.6, 61.7, 55.3, 55.2, 53.1, 36.8, 33.4, 33.0, 32.3, 31.9, 30.0, 29.6, 29.6, 29.5, 29.5, 29.4, 29.3, 29.3, 29.2, 28.9, 25.7, 22.7, 20.0, 14.1. HRMS (ESI) *m*/*z*: found [M+Na]^+^ 1196.7404, C_69_H_107_NO_12_S calcd for [M+Na]^+^ 1196.7406.


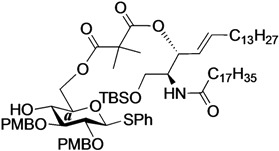


*Phenyl 6-O-{2-[(2S,3R,4E)-1-O-tert-butyldimethylsilyl-2-octadecanamido-4-octadecene-3-yloxy-1,3-diol]carbonylisobutanoyl}-2,3-di-O-p-methoxybenzyl-1-thio-β-d-glucopyranoside* (**14**). To a solution of **4** (55 mg, 107 µmol) in CH_2_Cl_2_ (2.1 mL) were added **8** (85 mg, 107 µmol), EDC·HCl (23 mg, 118 µmol) and DMAP (1.3 mg, 10.7 µmol) at 0 °C. After stirring for 5 h at rt as the reaction was monitored by TLC (2:1 diethylether–*n*-hexane), the mixture was diluted with CHCl_3_. The solution was then washed with H_2_O. The organic layer was subsequently dried over Na_2_SO_4_ and concentrated. The resulting residue was purified by silica gel column chromatography (1:5 EtOAc–*n*-hexane) to give **14** (70 mg, 51%). ^1^H-NMR (500 MHz, CDCl_3_) δ 7.54–6.85 (m, 13H, 3Ar), 6.70 (d, 1H, *J*_2,NH_ = 9.0 Hz, NH*^Cer^*), 5.75 (m, 1H, H-5*^Cer^*), 5.37 (m, 2H, H-4*^Cer^*, H-3*^Cer^*), 4.84 (d, 1H, *J*_gem_ = 11.0 Hz, ArC*H*_2_), 4.82 (d, 1H, *J*_gem_ = 10.0 Hz, ArC*H*_2_), 4.72 (d, 1H, ArC*H*_2_), 4.66 (d, 1H, ArC*H*_2_), 4.63 (d, 1H, *J*_1,2_ = 9.6 Hz, H-1a), 4.40 (dd, 1H, *J*_5,6_ = 4.0 Hz, *J*_gem_ = 11.9 Hz, H-6a), 4.34 (near dd, 1H, H-6'a), 4.15 (m, 1H, H-2*^Cer^*), 3.81–3.80 (2 s, 6H, 2OCH_3_), 3.68 (dd, 1H, *J*_1,2_ = 3.3 Hz, *J*_gem_ = 10.4 Hz, H-1*^Cer^*), 3.57 (dd, 1H, *J*_1',2_ = 4.3 Hz, H-1'*^Cer^*), 3.50–3.47 (m, 3H, H-3a, H-4a, H-5a), 3.40 (m, 1H, H-2a), 2.87 (br s, 1H, OHa), 2.28 (m, 2H, C(=O)CH_2_), 1.99 (m, 2H, H-6*^Cer^*, H-6'*^Cer^*), 1.59 (m, 2H, C(=O)CH_2_C*H*_2_), 1.45 (s, 6H, CH_3_CCH_3_), 1.25 (m, 50H, 25-CH_2_-), 0.88 (m, 15H, *t*-Bu, 2-CH_3_*^Cer^*), 0.05 (2 s, 6H, Si(CH_3_)_2_).


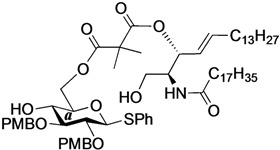


*Phenyl 6-O-{2-[(2S,3R,4E)-2-octadecanamido-4-octadecene-3-yloxy-1,3-diol]carbonylisobutanoyl}-2,3-di-O-p-methoxybenzyl-1-thio-β-d-glucopyranoside* (**15**). To a solution of **14** (62 mg, 48.1 µmol) in THF (481 µL) were added AcOH (8.6 µL, 144 µmol) and TBAF (144 µL, 144 µmol) at 0 °C. After stirring for 3.5 h at rt as the reaction was monitored by TLC (1:2 EtOAc–*n*-hexane), the mixture was diluted with CHCl_3_. The solution was then washed with satd aq NaHCO_3_. The organic layer was subsequently dried over Na_2_SO_4_ and concentrated. The resulting residue was purified by silica gel column chromatography (1:3 EtOAc–*n*-hexane) to give **15** (51 mg, 91%). ^1^H-NMR (500 MHz, CDCl_3_) δ 7.54–6.86 (m, 13H, 3Ar), 6.71 (d, 1H, *J*_2,NH_ = 8.6 Hz, NH*^Cer^*), 5.75 (m, 1H, H-5*^Cer^*), 5.41 (dd, 1H, *J*_3,4_ = 7.4 Hz, *J*_4,5_ = 15.4 Hz, H-4*^Cer^*), 5.29 (t, 1H, *J*_2,3_ = 7.4 Hz, H-3*^Cer^*), 4.83 (d, 1H, *J*_gem_ = 10.8 Hz, ArC*H*_2_), 4,82 (d, 1H, *J*_gem_ = 9.9 Hz, ArC*H*_2_), 4.72 (d, 1H, ArC*H*_2_), 4.66 (d, 1H, ArC*H*_2_), 4.64 (d, 1H, *J*_1,2_ = 9.2 Hz, H-1a), 4.39 (near d, 2H, H-6a, H-6'a), 4.08 (m, 1H, H-2*^Cer^*), 3.81–3.80 (2 s, 6H, 2OCH_3_), 3.55–3.46 (m, 5H, H-1*^Cer^*, H-1'*^Cer^*, H-3a, H-4a, H-5a), 3.40 (t, 1H, *J*_2,3_ = 9.2 Hz, H-2a), 3.32 (d, 1H, *J*_4,OH_ = 3.4 Hz, OHa), 2.83 (br s, 1H, OH*^Cer^*), 2.30 (m, 2H, C(=O)CH_2_), 2.01 (m, 2H, H-6*^Cer^*, H-6'*^Cer^*), 1.60 (m, 2H, C(=O)CH_2_C*H*_2_), 1.45 (2 s, 6H, CH_3_CCH_3_), 1.25 (m, 50H, 25-CH_2_-), 0.88 (m, 6H, 2-CH_3_*^Cer^*). HRMS (ESI) *m*/*z*: found [M+Na]^+^ 1196.7487, C_69_H_107_NO_12_S calcd for [M+Na]^+^ 1196.7406.


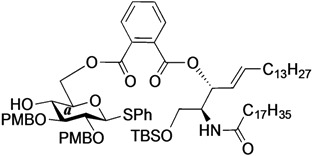


*Phenyl 6-O-{[(2S,3R,4E)-1-O-tert-butyldimethylsilyl-2-octadecanamido-4-octadecene-3-yloxy-1,3-diol]carbonylbenzoyl}-2,3-di-O-p-methoxybenzyl-1-thio-β-d-glucopyranoside* (**16**). To a solution of **4** (87 mg, 169 µmol) in CH_2_Cl_2_ (3.4 mL) were added **9** (140 mg, 169 µmol), EDC∙HCl (36 mg, 186 µmol) and DMAP (2.1 mg, 16.9 µmol) at 0 °C. After stirring for 5.5 h at rt as the reaction was monitored by TLC (1:1 EtOAc–*n*-hexane), the mixture was diluted with CHCl_3_. The solution was then washed with H_2_O. The organic layer was subsequently dried over Na_2_SO_4_ and concentrated. The resulting residue was purified by silica gel column chromatography (1:3 EtOAc–*n*-hexane) to give **16** (71 mg, 32%). [α]_D_ +4.5° (c 1.0, CHCl_3_); ^1^H-NMR (500 MHz, CDCl_3_) δ 7.77–6.83 (m, 17H, 4Ar), 5.93 (d, 1H, *J*_2,NH_ = 9.6 Hz, NH*^Cer^*), 5.77 (m, 1H, H-5*^Cer^*), 5.61 (t, 1H, *J*_2,3_ = *J*_3,4_ = 6.9 Hz, H-3*^Cer^*), 5.54 (dd, 1H, *J*_4,5_ = 15.4 Hz, H-4*^Cer^*), 4.82 (s, 2H, ArCH_2_), 4.79 (d, 1H, *J*_gem_ = 9.9 Hz, ArC*H*_2_), 4.68 (m, 3H, ArC*H*_2_, H-1a, H-6a), 4.57 (dd, 1H, *J*_5,6'_ = 1.9 Hz, *J*_gem_ = 12.0 Hz, H-6'a), 4.39 (m, 1H, H-2*^Cer^*), 4.11 (br s, 1H, OHa), 3.81–3.79 (2 s, 6H, 2OCH_3_), 3.71 (m, 2H, H-1*^Cer^*, H-4a), 3.63 (dd, 1H, *J*_1',2_ = 5.1 Hz, *J*_gem_ = 10.5 Hz, H-1'*^Cer^*), 3.57 (m, 2H, H-3a, H-5a), 3.45 (t, 1H, *J*_1,2_ = *J*_2,3_ = 9.2 Hz, H-2a), 2.15 (m, 2H, C(=O)CH_2_), 2.03 (m, 2H, H-6*^Cer^*, H-6'*^Cer^*), 1.55 (m, 2H, C(=O)CH_2_C*H*_2_), 1.25 (m, 50H, 25-CH_2_-), 0.88 (m, 15H, t-Bu, 2-CH_3_*^Cer^*), 0.04–0.03 (2 s, 6H, Si(CH_3_)_2_); ^13^C-NMR (125 MHz, CDCl_3_) δ 173.1, 166.9, 166.8, 159.4, 159.3, 136.2, 133.5, 133.1, 132.2, 131.8, 131.5, 131.0, 130.8, 130.7, 130.3, 129.8, 129.6, 129.2, 128.8, 127.5, 124.4, 113.9, 113.9, 113.8, 87.4, 85.8, 79.9, 77.8, 77.6, 75.8, 75.3, 75.0, 69.9, 64.3, 62.0, 55.3, 55.2, 52.0, 36.9, 32.4, 31.9, 29.7, 29.7, 29.5, 29.4, 29.4, 29.3, 29.2, 29.1, 25.8, 25.7, 22.7, 18.2, 14.1, −5.4, −5.6. HRMS (ESI) *m*/*z*: found [M+Na]^+^ 1344.8116, C_78_H_119_NO_12_SSi calcd for [M+Na]^+^ 1344.8114.


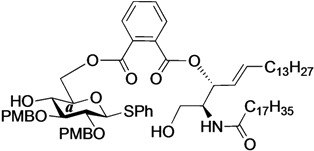


*Phenyl 6-O-{[(2S,3R,4E)-2-octadecanamido-4-octadecene-3-yloxy-1,3-diol]carbonylbenzoyl}-2,3-di-O-p-methoxybenzyl-1-thio-β-d-glucopyranoside* (**17**). To a solution of **16** (190 mg, 144 µmol) in THF (1.4 mL) were added AcOH (26 µL, 432 µmol) and TBAF (432 µL, 432 µmol) at 0 °C. After stirring for 4 h at rt as the reaction was monitored by TLC (1:1 EtOAc–*n*-hexane), the mixture was diluted with CHCl_3_. The solution was then washed with satd aq NaHCO_3_. The organic layer was subsequently dried over Na_2_SO_4_ and concentrated. The resulting residue was purified by silica gel column chromatography (1:2 EtOAc–*n*-hexane) to give **17** (156 mg, 90%). [α]_D_ +3.3° (c 0.3, CHCl_3_); ^1^H-NMR (500 MHz, CDCl_3_) δ 7.85–6.84 (m, 17H, 4Ar), 6.41 (d, 1H, *J*_2,NH_ = 9.2 Hz, NH*^Cer^*), 5.82 (m, 1H, H-5*^Cer^*), 5.61 (t, 1H, *J*_2,3_ = *J*_3,4_ = 6.6 Hz, H-3*^Cer^*), 5.47 (dd, 1H, *J*_4,5_ = 15.4 Hz, H-4*^Cer^*), 4.87–4.80 (m, 3H, ArC*H*_2_, H-6a), 4.73 (d, 1H, *J*_gem_ = 10.7 Hz, ArC*H*_2_), 4.68 (d, 1H, *J*_1,2_ = 9.2 Hz, H-1a), 4.65 (d, 1H, *J*_gem_ = 10.0 Hz, ArC*H*_2_), 4.48 (near d, 1H, H-6'a), 4.27 (m, 1H, H-2*^Cer^*), 3.80–3.78 (2 s, 6H, 2OCH_3_), 3.74 (br d, 1H, H-1*^Cer^*), 3.69 (s, 1H, OHa), 3.57–3.51 (m, 4H, H-4a, H-5a, H-3a, H-1'*^Cer^*), 3.42 (t, 1H, *J*_2,3_ = 9.2 Hz, H-2a), 2.85 (br s, 1H, OH*^Cer^*), 2.21 (m, 2H, C(=O)CH_2_), 2.03 (m, 2H, H-6*^Cer^*, H-6'*^Cer^*), 1.62 (m, 2H, C(=O)CH_2_C*H*_2_), 1.30 (m, 50H, 25-CH_2_-), 0.88 (m, 6H, 2-CH_3_*^Cer^*); ^13^C-NMR (125 MHz, CDCl_3_) δ 173.6, 167.3, 167.1, 159.4, 137.3, 133.5, 132.8, 132.0, 131.1, 130.4, 130.3, 130.1, 129.9, 129.8, 129.8, 129.7, 129.0, 128.9, 127.5, 124.3, 114.0, 113.9, 113.9, 87.6, 85.8, 80.0, 77.6, 77.4, 76.2, 75.5, 75.0, 69.2, 64.1, 61.6, 55.3, 55.2, 52.5, 36.7, 32.3, 31.9, 29.6, 29.6, 29.5, 29.5, 29.3, 29.2, 28.9, 25.7, 22.7, 14.1. HRMS (ESI) *m*/*z*: found [M+Na]^+^ 1230.7250, C_72_H_105_NO_12_S calcd for [M+Na]^+^ 1230.7250.


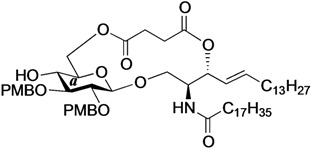


*(2,3-Di-O-p-methoxybenzyl-β-d-glucopyranosyl)-(1'→1)-(2S,3R,4E)-2-octadecanamido-4-octadecene-1,3-diol-3,6'-succinate* (**18β**). *Condition A*: To a mixture of **11** (32 mg, 27.6 µmol) in CH_2_Cl_2_ (5.5 mL) was added 4 Å molecular sieves (64 mg) at rt. After stirring for 1 h, the mixture was cooled to 0 °C. DMTST (45 mg, 82.8 µmol) was then added to the mixture at 0 °C. After stirring for 2 h at 0 °C as the reaction was monitored by TLC (1:2 acetone–*n*-hexane), the solution was diluted with CHCl_3_ and filtered through Celite. The filtrate was then washed with satd aq NaHCO_3_ and H_2_O. The organic layer was subsequently dried over Na_2_SO_4_, and concentrated. The residue was purified by silica gel column chromatography (1:5 acetone–*n*-hexane) to give **18** (19 mg, 67%, α:β = 1:1.7). *Condition B*: To a mixture of **11** (47 mg, 40.5 µmol) in acetonitrile/CH_2_Cl_2_ (2:1 8.1 mL) was added 3 Å molecular sieves (95 mg) at rt. After stirring for 1 h, the mixture was cooled to 0 °C. DMTST (65 mg, 121 µmol) was then added to the mixture at 0 °C. After stirring for 2 h at 0 °C as the reaction was monitored by TLC (1:2 acetone–*n*-hexane), the solution was diluted with CHCl_3_ and filtered through Celite. The filtrate was then washed with satd aq NaHCO_3_ and H_2_O. The organic layer was subsequently dried over Na_2_SO_4_, and concentrated. The residue was purified by silica gel column chromatography (1:5 acetone–*n*-hexane) to give **18** (33 mg, 77%, α:β = 1:8.2). **18β**: [α]_D_ −10.4° (c 0.5, CHCl_3_); ^1^H-NMR (500 MHz, CDCl_3_) δ 7.27–6.86 (m, 8H, 2Ar), 5.93 (d, 1H, *J*_2,NH_ = 9.2 Hz, NH*^Cer^*), 5.77 (m, 1H, H-5*^Cer^*), 5.55 (t, 1H, *J*_2,3_ = *J*_3,4_ = 6.3 Hz, H-3*^Cer^*), 5.30 (dd, 1H, *J*_4,5_ = 15.5 Hz, H-4*^Cer^*), 4.87 (d, 1H, *J*_gem_ = 11.4 Hz, ArCH_2_), 4.78 (d, 1H, *J*_gem_ = 10.8 Hz, ArC*H*_2_), 4.67–4.59 (m, 2H, H-6a, ArC*H*_2_), 4.55 (d, 1H, ArC*H*_2_), 4.40 (m, 1H, H-2*^Cer^*), 4.34 (d, 1H, *J*_1,2_ = 7.0 Hz, H-1a), 4.09 (dd, 1H, *J*_5,6'_ = 2.1 Hz, *J*_gem_ = 11.6 Hz, H-6'a), 3.97 (dd, 1H, *J*_1,2_ = 4.72 Hz, *J*_gem_ = 10.3 Hz, H-1*^Cer^*), 3.80 (s, 6H, 2OCH_3_), 3.77 (near d, 1H, H-1'*^Cer^*), 3.44 (td, 1H, *J*_4,5_ = 2.1 Hz, H-5a), 3.38–3.29 (m, 3H, H-2a, H-3a, H-4a), 2.79–2.61 (m, 4H, 2C(=O)CH_2_), 2.19 (d, 1H, *J*_4,OH_ = 1.8 Hz, OHa), 2.17 (m, 2H, C(=O)CH_2_*^Cer^*), 1.99 (m, 2H, H-6*^Cer^*, H-6'*^Cer^*), 1.61 (m, 2H, C(=O)CH_2_C*H*_2_), 1.25 (m, 50H, 25-CH_2_-), 0.88 (m, 6H, 2-CH_3_*^Cer^*); ^13^C-NMR (125 MHz, CDCl_3_) δ 173.1, 172.6, 169.9, 159.4, 159.3, 136.5, 130.4, 130.1, 129.8, 129.6, 124.1, 114.0, 113.8, 102.8, 83.2, 81.0, 74.7, 74.1, 73.7, 73.4, 70.8, 65.0, 63.7, 55.2, 50.4, 36.8, 32.2, 31.9, 29.7, 29.7, 29.7, 29.6, 29.6, 29.5, 29.5, 29.4, 29.3, 29.2, 29.0, 28.8, 25.7, 22.6, 14.1. HRMS (ESI) *m*/*z*: found [M+Na]^+^ 1072.7060, C_62_H_99_NO_12_ calcd for [M+Na]^+^ 1072.7059.


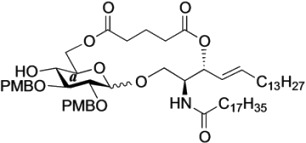


*(2,3-Di-O-p-methoxybenzyl-d-glucopyranosyl)-(1'→1)-(2S,3R,4E)-2-octadecanamido-4-octadecene-1,3-diol-3,6´-glutarate* (**19**). *Condition A*: To a mixture of **13** (27 mg, 23.0 µmol) in CH_2_Cl_2_ (4.6 mL) was added 4 Å molecular sieves (55 mg) at rt. After stirring for 1 h, the mixture was cooled to 0 °C. DMTST (37 mg, 69.0 µmol) was then added to the mixture at 0 °C. After stirring for 3 h at 0 °C as the reaction was monitored by TLC (1:2 acetone–*n*-hexane), the solution was diluted with CHCl_3_ and filtered through Celite. The filtrate was then washed with satd aq NaHCO_3_ and H_2_O. The organic layer was subsequently dried over Na_2_SO_4_, and concentrated. The residue was purified by silica gel column chromatography (1:5 acetone–*n*-hexane) to give **19** (8.3 mg, 34%, α:β = 1:2.0). *Condition B*: To a mixture of **13** (29 mg, 24.7 µmol) in acetonitrile/CH_2_Cl_2_ (2:1 4.9 mL) was added 3 Å molecular sieves (60 mg) at rt. After stirring for 1 h, the mixture was cooled to 0 °C. DMTST (40 mg, 74.1 µmol) was then added to the mixture at 0 °C. After stirring for 20 h at 0 °C as the reaction was monitored by TLC (1:2 acetone–*n*-hexane), the solution was diluted with CHCl_3_ and filtered through Celite. The filtrate was then washed with satd aq NaHCO_3_ and H_2_O. The organic layer was subsequently dried over Na_2_SO_4_, and concentrated. The residue was purified by silica gel column chromatography (1:5 acetone–*n*-hexane) to give **19** (10.4 mg, 40%, α:β = 1:7.7). **19β**: ^1^H-NMR (500 MHz, CDCl_3_) δ 7.29–6.86 (m, 8H, 2Ar), 5.79 (m, 1H, H-5*^Cer^*), 5.73 (d, 1H, *J*_2,NH_ = 8.7 Hz, NH*^Cer^*), 5.32 (m, 2H, H-4*^Cer^*, H-3*^Cer^*), 4.89 (d, 1H, *J*_gem_ = 11.3 Hz, ArC*H*_2_), 4.79 (d, 1H, *J*_gem_ = 10.9 Hz, ArC*H*_2_), 4.68 (d, 1H, ArC*H*_2_), 4.58 (m, 2H, H-6a, ArC*H*_2_), 4.37 (d, 1H, *J*_1,2_ = 7.3 Hz, H-1a), 4.27 (m, 1H, H-2*^Cer^*), 4.02 (dd, 1H, *J*_5,6´_ = 4.5 Hz, *J*_gem_ = 11.8 Hz, H-6´a), 3.96 (dd, 1H, *J*_1,2_ = 4.6 Hz, *J*_gem_ = 9.9 Hz, H-1*^Cer^*), 3.80 (2 s, 6H, 2OCH_3_), 3.69 (dd, 1H, *J*_1',2_ = 3.0 Hz, H-1'*^Cer^*), 3.44 (m, 2H, H-4a, H-5a), 3.37 (m, 2H, H-2a, H-3a), 2.57–2.29 (m, 4H, 2C(=O)CH_2_), 2.13 (s, 1H, OHa), 2.09–1.85 (m, 6H, C(=O)CH_2_*^Cer^*, H-6*^Cer^*, H-6'*^Cer^*, -CH_2_-), 1.54 (m, 2H, C(=O)CH_2_C*H_2_*), 1.23 (m, 50H, 25-CH_2_-*^Cer^*), 0.88 (m, 6H, 2-CH_3_*^Cer^*); ^13^C-NMR (125 MHz, CDCl_3_) δ 172.9, 172.7, 171.3, 159.5, 159.4, 137.2, 130.4, 130.3, 129.6, 129.5, 125.1, 114.1, 114.0, 113.9, 102.7, 83.2, 81.7, 77.6, 74.8, 74.5, 72.6, 72.4, 69.9, 67.0, 62.5, 55.3, 55.2, 50.9, 36.8, 33.0, 32.5, 32.3, 31.9, 30.0, 29.7, 29.6, 29.6, 29.5, 29.4, 29.4, 29.2, 28.9, 25.7, 22.7, 19.6, 14.1. HRMS (ESI) *m*/*z*: found [M+Na]^+^ 1086.7216, C_63_H_101_NO_12_ calcd for [M+Na]^+^ 1086.7216.


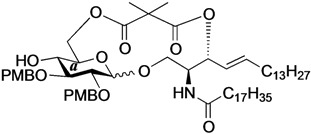


*(2,3-Di-O-p-methoxybenzyl-d-glucopyranosyl)-(1'→1)-(2S,3R,4E)-2-octadecanamido-4-octadecene-1,3-diol-3,6´-(2,2-dimethylmalonate)* (**20**). *Condition A*: To a mixture of **15** (24 mg, 20.4 µmol) in CH_2_Cl_2_ (4.1 mL) was added 4 Å molecular sieves (50 mg) at rt. After stirring for 1 h, the mixture was cooled to 0 °C. DMTST (33 mg, 61.2 µmol) was then added to the mixture at 0 °C. After stirring for 2 h at 0 °C as the reaction was monitored by TLC (1:2 acetone–*n*-hexane), the solution was diluted with CHCl_3_ and filtered through Celite. The filtrate was then washed with satd aq NaHCO_3_ and H_2_O. The organic layer was subsequently dried over Na_2_SO_4_, and concentrated. The residue was purified by silica gel column chromatography (1:5 acetone–*n*-hexane) to give **20** (16 mg, 75%, α:β = 1:2.4). *Condition B*: To a mixture of **15** (26 mg, 22.2 µmol) in acetonitrile/CH_2_Cl_2_ (2:1 4.5 mL) was added 3 Å molecular sieves (55 mg) at rt. After stirring for 1 h, the mixture was cooled to 0 °C. DMTST (36 mg, 66.6 µmol) was then added to the mixture at 0 °C. After stirring for 2 h at 0 °C as the reaction was monitored by TLC (1:2 acetone–*n*-hexane), the solution was diluted with CHCl_3_ and filtered through Celite. The filtrate was then washed with satd aq NaHCO_3_ and H_2_O. The organic layer was subsequently dried over Na_2_SO_4_, and concentrated. The residue was purified by silica gel column chromatography (1:5 acetone–*n*-hexane) to give **20** (18 mg, 76%, α:β = 1:9.1). **20β**: ^1^H-NMR (500 MHz, CDCl_3_) δ 7.24–6.85 (m, 8H, 2Ar), 6.55 (d, 1H, *J*_2,NH_ = 7.0 Hz, NH*^Cer^*), 5.75 (m, 1H, H-5*^Cer^*), 5.59 (t, 1H, *J*_2,3_ = *J*_3,4_ = 6.4 Hz, H-3*^Cer^*), 5.33 (dd, 1H, *J*_4,5_ = 15.4 Hz, H-4*^Cer^*), 4.81 (d, 1H, *J*_gem_ = 11.5 Hz, ArC*H*_2_), 4.66 (d, 1H, *J*_gem_ = 11.0 Hz, ArC*H*_2_), 4.59 (dd, 1H, *J*_5,6_ = 5.2 Hz, *J*_gem_ = 11.7 Hz, H-6a), 4.55 (d, 1H, ArC*H*_2_), 4.54 (d, 1H, ArC*H*_2_), 4.44 (m, 2H, H-1a, H-6'a), 4.56 (dd, 1H, *J*_gem_ = 11.9 Hz, *J*_1,2_ = 2.1 Hz, H-1*^Cer^*), 3.89 (m, 1H, H-2*^Cer^*), 3.59 (m, 2H, H-1'*^Cer^*, H-5a), 3.48 (t, 1H, *J*_3,4_ = *J*_4,5_ = 7.3 Hz, H-4a), 3.44 (t, 1H, *J*_1,2_ = *J*_2,3_ = 7.3 Hz, H-2a), 3.36 (t, 1H, H-3a), 2.44 (s, 1H, OHa), 2.30 (m, 2H, C(=O)CH_2_), 2.00 (m, 2H, H-6*^Cer^*, H-6'*^Cer^*), 1.60 (m, 2H, C(=O)CH_2_C*H*_2_), 1.41–1.40 (2 s, 6H, CH_3_CCH_3_), 1.25 (m, 50H, 25-CH_2_-), 0.88 (m, 6H, 2-CH_3_*^Cer^*); ^13^C-NMR (125 MHz, CDCl_3_) δ 175.0, 172.4, 170.7, 159.5, 159.4, 136.2, 130.3, 130.1, 129.7, 129.7, 129.6, 129.5, 129.0, 127.6, 127.1, 124.6, 114.2, 114.0, 114.0, 113.9, 103.6, 82.2, 80.5, 74.1, 73.3, 72.8, 72.2, 70.4, 69.8, 62.7, 55.3, 54.6, 50.8, 50.7, 34.5, 32.3, 29.6, 29.5, 29.5, 29.3, 29.3, 29.2, 29.2, 29.0, 28.9, 25.0, 23.7, 22.7, 21.8, 14.1. HRMS (ESI) *m*/*z*: found [M+Na]^+^ 1086.7217, C_63_H_101_NO_12_ calcd for [M+Na]^+^ 1086.7216.


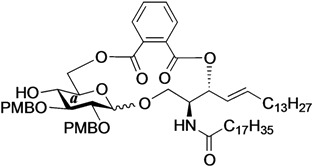


*(2,3-Di-O-p-methoxybenzyl-d-glucopyranosyl)-(1'→1)-(2S,3R,4E)-2-octadecanamido-4-octadecene-1,3-diol-3,6'-phthalate* (**21**). *Condition A*: To a mixture of **17** (50 mg, 41.4 µmol) in CH_2_Cl_2_ (8.3 mL) was added 4 Å molecular sieves (100 mg) at rt. After stirring for 1 h, the mixture was cooled to 0 °C. DMTST (67 mg, 124 µmol) was then added to the mixture at 0 °C. After stirring for 1 h at 0 °C as the reaction was monitored by TLC (1:2 acetone–*n*-hexane), the solution was diluted with CHCl_3_ and filtered through Celite. The filtrate was then washed with satd aq NaHCO_3_ and H_2_O. The organic layer was subsequently dried over Na_2_SO_4_, and concentrated. The residue was purified by silica gel column chromatography (1:5 acetone–*n*-hexane) to give **21** (24 mg, 53%, α:β = 1:2.0). *Condition B*: To a mixture of **17** (45 mg, 37.3 µmol) in acetonitrile/CH_2_Cl_2_ (2:1 11.3 mL) was added 3 Å molecular sieves (90 mg) at rt. After stirring for 1 h, the mixture was cooled to 0 °C. DMTST (60 mg, 112 µmol) was then added to the mixture at 0 °C. After stirring for 1 h at 0 °C as the reaction was monitored by TLC (1:2 acetone–*n*-hexane), the solution was diluted with CHCl_3_ and filtered through Celite. The filtrate was then washed with satd aq NaHCO_3_ and H_2_O. The organic layer was subsequently dried over Na_2_SO_4_, and concentrated. The residue was purified by silica gel column chromatography (1:5 acetone–*n*-hexane) to give **21** (29 mg, 71%, α:β = 1:5.2). **21β**: ^1^H-NMR (500 MHz, CDCl_3_) δ 7.75–6.86 (m, 12H, 3Ar), 6.18 (br d, 1H, *J*_2,NH_ = 9.8 Hz, NH*^Cer^*), 5.88 (m, 1H, H-5*^Cer^*), 5.67 (m, 1H, H-3*^Cer^*), 5.41 (dd, 1H, *J*_3,4_ = 7.8 Hz, *J*_4,5_ = 15.3 Hz, H-4*^Cer^*), 4.90 (d, 1H, *J*_gem_ = 11.4 Hz, ArC*H*_2_), 4.81 (d, 1H, *J*_gem_ = 10.7 Hz, ArC*H*_2_), 4.64 (d, 1H, ArC*H*_2_), 4.56 (d, 1H, ArC*H*_2_), 4.53–4.47 (m, 3H, H-6a, H-6'a, H-2*^Cer^*), 4.33 (d, 1H, *J*_1,2_ = 7.4 Hz, H-1a), 3.97 (dd, 1H, *J*_1,2_ = 4.4 Hz, *J*_gem_ = 10.3 Hz, H-1*^Cer^*), 3.90 (near dd, 1H, *J*_1',2_ = 5.4 Hz, H-1'*^Cer^*), 3.82–3.78 (m, 7H, H-4a, 2OCH_3_), 3.50 (m, 1H, H-5a), 3.43–3.36 (m, 2H, H-2a, H-3a), 2.22 (m, 2H, C(=O)CH_2_), 2.16 (s, 1H, OHa), 2.00 (m, 2H, H-6*^Cer^*, H-6'*^Cer^*), 1.64 (m, 2H, C(=O)CH_2_C*H_2_*), 1.25 (m, 50H, 25-CH_2_-), 0.88 (m, 6H, 2-CH_3_*^Cer^*); ^13^C-NMR (125 MHz, CDCl_3_) δ 172.8, 159.5, 159.4, 131.5, 130.9, 130.4, 130.2, 129.8, 129.7, 129.6, 129.0, 124.4, 124.1, 114.1, 114.1, 114.0, 113.9, 103.1, 83.4, 81.3, 77.6, 75.1, 74.8, 74.3, 70.9, 55.3, 51.8, 50.2, 37.0, 32.3, 31.9, 29.7, 29.7, 29.7, 29.6, 29.6, 29.5, 29.5, 29.4, 29.2, 28.8, 25.9, 25.6, 22.7, 14.1. HRMS (ESI) *m*/*z*: found [M+Na]^+^ 1120.7060, C_66_H_99_NO_12_ calcd for [M+Na]^+^ 1120.7059.


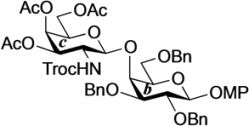


*4-Methoxyphenyl 3,4,6-tri-O-acetyl-2-deoxy-2-(2,2,2-trichloroethoxycarbamoyl)-β-d-galactopyranosyl-(1→4)-2,3,6-tri-O-benzyl-β-d-galactopyranoside* (**24**). To a mixture of **22** (80 mg, 144 µmol) and **23** (82 mg, 144 µmol) in CH_2_Cl_2_ (1.4 mL) was added 4 Å molecular sieves (320 mg) at rt. After stirring for 1 h, the mixture was cooled to 0 °C. NIS (49 mg, 216 µmol) and TfOH (1.9 µL, 21.6 µmol) were then added to the mixture at 0 °C. After stirring for 2 h at 0 °C as the reaction was monitored by TLC (4:1 toluene–EtOAc), the reaction was quenched by the addition of satd aq NaHCO_3_. The solution was diluted with CHCl_3_ and filtered through Celite. The filtrate was then washed with satd aq Na_2_S_2_O_3_ and brine. The organic layer was subsequently dried over Na_2_SO_4_, and concentrated. The residue was purified by silica gel column chromatography (10:1 toluene–EtOAc) to give **24** (126 mg, 86%). [α]_D_ −5.6° (c 0.8, CHCl_3_); ^1^H-NMR (600 MHz, CDCl_3_) δ 7.39–7.26 (m, 15H, 3 Ph), 7.04–6.79 (m, 4H, Ar), 5.65 (d, 1H, *J*_2,NH_ = 6.9 Hz, NHc), 5.27 (d, 1H, *J*_3,4_ = 2.8 Hz, H-4c), 5.04 (d, 1H, *J*_gem_ = 11.0 Hz, PhC*H*_2_), 4.96 (d, 1H, *J*_gem_ = 11.0 Hz, PhC*H*_2_), 4.91 (d, 1H, *J*_gem_ = 12.4 Hz, PhC*H*_2_), 4.84 (d, 2H, *J*_1,2_ = 7.6 Hz, H-1b, PhC*H*_2_), 4.70 (m, 2H, H-3c, H-1c), 4.64 (d, 1H, PhC*H*_2_), 4.55 (q, 2H, *J*_gem_ = 11.7 Hz, OCH_2_CCl_3_), 4.41 (d, 1H, PhC*H*_2_), 4.10 (dd, 1H, *J*_5,6_ = 7.6 Hz, *J*_gem_ = 11.0 Hz, H-6c), 4.06 (d, 1H, *J*_3,4_ = 2.8 Hz, H-4b), 4.04 (dd, 1H, *J*_5,6'_ = 6.2 Hz, H-6'c), 3.92 (m, 2H, H-2b, H-2c), 3.80 (dd, 1H, *J*_5,6_ = 5.8 Hz, *J*_gem_ = 10.0 Hz, H-6b), 3.77 (s, 3H, OCH_3_), 3.76–3.71 (m, 2H, H-6'b, H-5c), 3.66 (m, 2H, H-3b, H-5b), 2.14–1.94 (3 s, 9H, 3Ac); ^13^C-NMR (150 MHz, CDCl_3_) δ 170.3, 170.2, 155.3, 154.3, 151.4, 138.2, 138.1, 137.2, 129.0, 128.7, 128.5, 128.4, 128.2, 128.0, 127.8, 127.7, 127.6, 125.3, 118.6, 114.5, 102.9, 101.9, 95.8, 81.6, 79.6, 75.7, 75.3, 74.6, 74.3, 73.6, 73.5, 71.7, 70.9, 69.1, 66.5, 61.1, 55.6, 52.7, 20.6, 20.5. HRMS (ESI) *m*/*z*: found [M+Na]^+^ 1040.2402, C_49_H_54_Cl_3_NO_16_ calcd for [M+Na]^+^ 1040.2400.


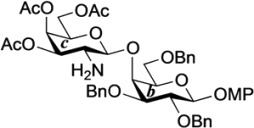


*4-Methoxyphenyl 3,4,6-tri-O-acetyl-2-amino-2-deoxy-β-d-galactopyranosyl-(1→4)-2,3,6-tri-O-benzyl-β-d-galactopyranoside* (**25**). To a solution of **24** (100 mg, 98.3 µmol) in acetonitrile/AcOH (4:1, 3.3 mL) was added Zn (500 mg) at rt. After stirring for 30 min at rt as the reaction was monitored by TLC (1:1 toluene–EtOAc), the solution was diluted with EtOAc and filtered through Celite. The filtrate was then washed with satd aq Na_2_CO_3_ and brine. The organic layer was subsequently dried over Na_2_SO_4_, and concentrated. The residue was purified by silica gel column chromatography (1.5:1 toluene–EtOAc) to give **25** (81 mg, 98%). [α]_D_ −12.3° (c 0.4, CHCl_3_); ^1^H-NMR (600 MHz, CDCl_3_) δ 7.36–7.24 (m, 15H, 3Ph), 7.06–6.78 (m, 4H, Ar), 5.29 (d, 1H, *J*_3,4_ = 2.1 Hz, H-4c), 5.01 (d, 1H, *J*_gem_ = 11.0 Hz, PhC*H*_2_), 4.87 (d, 1H, *J*_1,2_ = 7.6 Hz, H-1b), 4.83 (d, 1H, *J*_gem_ = 11.7 Hz, PhC*H*_2_), 4.82 (d, 1H, *J*_gem_ = 11.6 Hz, PhC*H*_2_), 4.69 (d, 1H, PhC*H*_2_), 4.66 (dd, 1H, *J*_2,3_ = 10.7 Hz, H-3c), 4.55 (s, 2H, PhC*H*_2_), 4.49 (d, 1H, *J*_1,2_ = 8.2 Hz, H-1c), 4.08 (dd, 1H, *J*_5,6_ = 7.6 Hz, *J*_gem_ = 11.0 Hz, H-6c), 4.04 (d, 1H, *J*_3,4_ = 2.8 Hz, H-4b), 4.02 (dd, 1H, *J*_5,6'_ = 6.2 Hz, H-6'c), 3.94 (dd, 1H, *J*_1,2_ = 7.6 Hz, *J*_2,3_ = 9.6 Hz, H-2b), 3.80 (dd, 1H, *J*_5,6_ = 4.8 Hz, *J*_gem_ = 10.3 Hz, H-6b), 3.76 (s, 3H, OCH_3_), 3.76–3.73 (m, 2H, H-6'b, H-5c), 3.66 (m, 1H, H-5b), 3.59 (dd, 1H, H-3b), 3.15 (dd, 1H, H-2c), 2.09–2.01 (3 s, 9H, 3Ac); ^13^C-NMR (150 MHz, CDCl_3_) δ 170.4, 170.3, 170.2, 155.2, 151.5, 138.2, 138.1, 137.9, 129.0, 128.6, 128.4, 128.3, 128.2, 128.0, 127.8, 127.6, 127.5, 125.3, 118.3, 114.5, 105.1, 102.9, 81.0, 78.8, 75.2, 74.8, 74.1, 73.9, 73.6, 73.6, 70.5, 69.8, 66.3, 61.5, 55.6, 51.9, 21.4, 20.8, 20.7, 20.6. HRMS (ESI) *m*/*z*: found [M+Na]^+^ 866.3358, C_46_H_53_NO_14_ calcd for [M+Na]^+^ 866.3358.


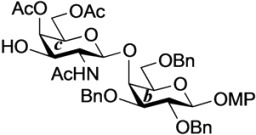


*4-Methoxyphenyl 2-acetamido-4,6-di-O-acetyl-2-deoxy-β-d-galactopyranosyl-(1→4)-2,3,6-tri-O-benzyl-β-d-galactopyranoside* (**26**). Compound **25** (270 mg, 320 µmol) was dissolved in 1,4-dioxane/AcOH (4:1, 32 mL) and the solution was stirred for 50 h at 60 °C as the reaction was monitored by TLC (1:1.5 toluene–EtOAc). Dilution of the mixture with CHCl_3_ provided a solution, which was then washed with satd aq Na_2_CO_3_. The organic layer was subsequently dried over Na_2_SO_4_, and concentrated. The residue was purified by silica gel column chromatography (2:1 toluene–EtOAc) to give **26** (218 mg, 81%). [α]_D_ −3.5° (c 0.6, CHCl_3_); ^1^H-NMR (600 MHz, CDCl_3_) δ 7.38–7.24 (m, 15H, 3Ph), 7.17 (d, 1H, *J*_2,NH_ = 3.4 Hz, NHc), 7.05–6.78 (m, 4H, Ar), 5.91 (d, 1H, *J*_3,OH_ = 1.4 Hz, OHc), 5.27 (d, 1H, *J*_3,4_ = 3.4 Hz, H-4c), 5.12 (d, 1H, *J*_gem_ = 11.0 Hz, PhC*H*_2_), 4.88 (d, 1H, *J*_1,2_ = 7.6 Hz, H-1b), 4.87 (d, 1H, *J*_gem_ = 9.6 Hz, PhC*H*_2_), 4.73 (d, 2H, PhC*H*_2_), 4.56 (q, 2H, *J*_gem_ = 11.7 Hz, PhC*H*_2_), 4.46 (d, 1H, *J*_1,2_ = 8.2 Hz, H-1c), 4.15 (dd, 1H, *J*_5,6_ = 6.5 Hz, *J*_gem_ = 11.3 Hz, H-6c), 4.05 (m, 2H, H-6'c, H-4b), 3.89–3.83 (m, 3H, H-2b, H-2c, H-6b), 3.77 (s, 3H, OCH_3_), 3.76–3.69 (m, 4H, H-6'b, H-3b, H-5b, H-5c), 3.53 (br d, 1H, H-3c), 2.14–1.61 (3 s, 9H, 3Ac); ^13^C-NMR (150 MHz, CDCl_3_) δ 173.9, 170.5, 170.3, 155.4, 151.2, 138.1, 138.0, 136.4, 129.1, 129.0, 128.8, 128.5, 128.4, 128.2, 128.0, 127.9, 127.7, 127.5, 118.5, 114.5, 102.9, 102.7, 81.6, 79.9, 75.7, 75.3, 74.3, 73.6, 71.3, 69.3, 67.9, 61.9, 55.8, 55.6, 29.7, 22.3, 20.8, 20.7. HRMS (ESI) *m*/*z*: found [M+Na]^+^ 866.3358, C_46_H_53_NO_14_ calcd for [M+Na]^+^ 866.3358.


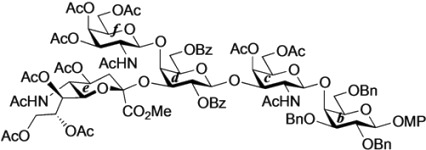


*4-Methoxyphenyl 2-acetamido-3,4,6-tri-O-acetyl-2-deoxy-β-d-galactopyranosyl-(1→4)-{[methyl 5-acetamido-4,7,8,9-tetra-O-acetyl-3,5-dideoxy-d-glycero-α-d-galacto-2-nonulopyranosylonate]-(2→3)}-2,6-di-O-benzoyl-β-d-galactopyranosyl-(1→3)-2-acetamido-4,6-di-O-acetyl-2-deoxy-β-d-galactopyranosyl-(1→4)-2,3,6-tri-O-benzyl-β-d-galactopyranoside* (**29**). To a mixture of **28** (180 mg, 135 µmol) and **26** (114 mg, 135 µmol) in CH_2_Cl_2_ (4.5 mL) was added 4 Å molecular sieves (300 mg) at rt. After stirring for 1 h, the mixture was cooled to 0 °C. TMSOTf (2.4 µL, 13.5 µmol) was then added to the mixture at 0 °C. After stirring for 2 h at 0 °C as the reaction was monitored by TLC (1:1 CHCl_3_–acetone), the reaction was quenched by the addition of satd aq NaHCO_3_. The solution was diluted with CHCl_3_ and filtered through Celite. The filtrate was then washed with satd aq NaHCO_3_ and brine. The organic layer was subsequently dried over Na_2_SO_4_, and concentrated. The residue was purified by silica gel column chromatography (40:1 CHCl_3_–MeOH) to give **29** (203 mg, 75%). [α]_D_ −7.7° (c 0.2, CHCl_3_); ^1^H-NMR (600 MHz, DMSO-*d*_6_) δ 8.00–7.19 (m, 25H, 5Ph), 7.57 (d, 1H, *J*_5,NH_ = 8.9 Hz, NHe), 7.20 (d, 1H, *J*_2,NH_ = 8.2 Hz, NHc), 6.96–6.78 (m, 4H, Ar), 6.78 (d, 1H, *J*_2,NH_ = 6.9 Hz, NHf), 5.32 (d, 1H, *J*_3,4_ = 3.5 Hz, H-4c), 5.29–5.25 (m, 2H, H-8e, H-3f), 5.23 (d, 1H, *J*_3,4_ = 2.7 Hz, H-4f), 5.14–5.09 (m, 2H, H-2d, H-7e), 4.90 (d, 1H, *J*_1,2_ = 7.6 Hz, H-1d), 4.85 (d, 2H, *J*_1,2_ = 7.6 Hz, H-1b, H-1f), 4.78 (m, 1H, H-4e), 4.74 (d, 1H, *J*_gem_ = 11.7 Hz, PhC*H*_2_), 4.68 (m, 2H, H-1c, PhC*H*_2_), 4.59 (m, 2H, H-3d, PhC*H*_2_), 4.52 (d, 1H, *J*_gem_ = 12.4 Hz, PhC*H*_2_), 4.47 (m, 2H, H-6d, PhC*H*_2_), 4.41 (d, 1H, *J*_gem_ = 12.4 Hz, PhC*H*_2_), 4.28 (m, 1H, H-3c), 4.25 (dd, 1H, *J*_5,6'_ = 5.5 Hz, *J*_gem_ = 11.0 Hz, H-6'd), 4.08–4.00 (m, 3H, H-6c, H-6f, H-9e), 3.98–3.91 (m, 4H, H-6'c, H-6'f, H-9'e, H-5d), 3.86–3.72 (m, 8H, H-4d, H-5c, H-5f, H-2c, H-2f, H-6e, H-6b, H-5e), 3.75–3.68 (2 s, 6H, 2OCH_3_), 3.66 (near t, 1H, H-2b), 3.59–3.53 (m, 4H, H-3b, H-4b, H-5b, H-6'b), 2.30 (dd, 1H, *J*_3eq,4_ = 4.8 Hz, *J*_gem_ = 13.1 Hz, H-3e*eq*), 1.80 (near t, 1H, H-3e*ax*), 2.08–1.64 (12 s, 36H, 12 Ac); ^13^C-NMR (150 MHz, CDCl_3_) δ 171.3, 170.7, 170.6, 170.5, 170.4, 170.4, 170.2, 169.9, 169.8, 168.1, 166.0, 164.3, 155.1, 151.5, 138.4, 137.9, 133.2, 133.1, 130.0, 129.5, 128.6, 128.5, 128.4, 128.3, 128.0, 127.7, 127.5, 118.3, 114.4, 102.8, 100.7, 100.5, 100.0, 98.3, 80.7, 79.2, 75.3, 74.9, 74.1, 74.0, 73.8, 73.5, 73.2, 72.1, 72.0, 70.8, 70.3, 70.1, 70.0, 69.3, 68.7, 67.4, 66.9, 66.5, 63.7, 62.7, 62.1, 61.4, 55.6, 54.2, 53.1, 51.8, 49.2, 36.1, 31.9, 29.7, 29.3, 23.4, 23.1, 22.7, 21.1, 20.8, 20.7, 20.7, 20.7, 20.5, 20.4, 14.1. HRMS (ESI) *m*/*z*: found [M+Na]^+^ 2038.7055, C_100_H_117_N_3_O_41_ calcd for [M+Na]^+^ 2038.7055.


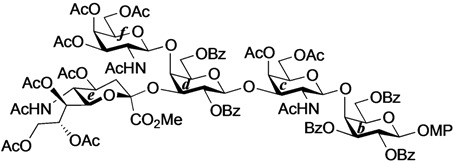


*4-Methoxyphenyl 2-acetamido-3,4,6-tri-O-acetyl-2-deoxy-β-d-galactopyranosyl-(1→4)-{[methyl 5-acetamido-4,7,8,9-tetra-O-acetyl-3,5-dideoxy-d-glycero-α-d-galacto-2-nonulopyranosylonate]-(2→3)}-2,6-di-O-benzoyl-β-d-galactopyranosyl-(1→3)-2-acetamido-4,6-di-O-acetyl-2-deoxy-β-d-galactopyranosyl-(1→4)-2,3,6-tri-O-benzoyl-β-d-galactopyranoside* (**30**). To a solution of **29** (215 mg, 107 µmol) in EtOH (10.7 mL) was added Pd(OH)_2_/C (20%, 215 mg). After stirring for 16 h at rt under a hydrogen atmosphere as the reaction was monitored by TLC (10:1 CHCl_3_–MeOH), the mixture was filtered through Celite. The filtrate was concentrated and the crude residue obtained was exposed to high vacuum for 3 h. The resulting residue was then dissolved in pyridine (535 µL). Benzoic anhydride (145 mg, 642 µmol) and DMAP (7.8 mg, 64.2 µmol) were added to the mixture at 0 °C. After stirring for 40 min at rt as the reaction was monitored by TLC (15:1 CHCl_3_–MeOH), the reaction was quenched by the addition of MeOH at 0 °C. The mixture was co-evaporated with toluene and the residue was then diluted with CHCl_3_, and washed with 2 M HCl, H_2_O and satd aq NaHCO_3_. The organic layer was subsequently dried over Na_2_SO_4_, and concentrated. The resulting residue was purified by silica gel column chromatography (25:1 CHCl_3_–MeOH) to give **30** (180 mg, 82%). [α]_D_ +6.3° (c 0.4, CHCl_3_); ^1^H-NMR (600 MHz, DMSO-*d*_6_) δ 8.01–7.35 (m, 26H, 5Ph, NHe), 7.11 (br d, 1H, *J*_2,NH_ = 5.5 Hz, NHc), 7.01 (d, 1H, *J*_2,NH_ = 8.2 Hz, NHf), 6.85–6.62 (m, 4H, Ar), 5.60 (t, 1H, *J*_1,2_ = *J*_2,3_ = 7.6 Hz, H-2b), 5.52 (dd, 1H, *J*_3,4_ = 2.8 Hz, H-3b), 5.39 (d, 1H, H-1b), 5.30 (d, 1H, *J*_3,4_ = 2.7 Hz, H-4c), 5.25 (m, 3H, H-3f, H-8e, H-4f), 5.12 (m, 2H, H-2d, H-7e), 4.87 (m, 2H, H-1d, H-1f), 4.81 (m, 1H, H-4e), 4.74 (d, 1H, *J*_1,2_ = 8.3 Hz, H-1c), 4.59 (d, 1H, *J*_2,3_ = 10.3 Hz, H-3d), 4.49–4.41 (m, 5H, H-6b, H-4b, H-6'b, H-6d, H-5b), 4.29 (br dd, 1H, *J*_2,3_ = 10.3 Hz, H-3c), 4.24 (dd, 1H, *J*_5,6'_ = 5.5 Hz, *J*_gem_ = 11.0 Hz, H-6'd), 4.06 (m, 2H, H-6f, H-9e), 3.98–3.93 (m, 3H, H-6'f, H-9'e, H-6c), 3.90–3.72 (m, 7H, H-4d, H-5d, H-5f, H-2f, H-6e, H-5e, H-6'c), 3.75 (s, 3H, OCH_3_), 3.67 (m, 1H, H-5c), 3.62 (s, 3H, OCH_3_), 3.60 (m, 1H, H-2c), 2.26 (near dd, 1H, H-3e*eq*), 2.09–1.75 (m, 37H, H-3e*ax*, 12Ac); ^13^C-NMR (150 MHz, CDCl_3_) δ 171.1, 170.6, 170.5, 170.5, 170.5, 170.3, 170.2, 169.9, 169.7, 168.1, 166.3, 166.1, 166.0, 165.3, 164.3, 155.5, 151.2, 133.6, 133.4, 133.1, 132.9, 130.1, 130.0, 130.0, 129.8, 129.7, 129.6, 129.5, 129.4, 128.6, 128.5, 128.4, 128.3, 118.7, 114.3, 100.9, 100.8, 100.4, 98.2, 97.8, 74.7, 74.0, 73.4, 72.7, 72.0, 71.9, 71.2, 70.6, 70.1, 70.0, 69.7, 69.4, 68.8, 67.3, 67.0, 66.5, 63.7, 63.5, 62.8, 62.3, 61.4, 55.5, 53.1, 52.1, 52.0, 49.3, 36.3, 29.7, 23.4, 23.2, 21.1, 20.9, 20.8, 20.8, 20.7, 20.5, 20.4. HRMS (ESI) *m*/*z*: found [M+Na]^+^ 2080.6434, C_100_H_111_N_3_O_44_ calcd for [M+Na]^+^ 2080.6433.


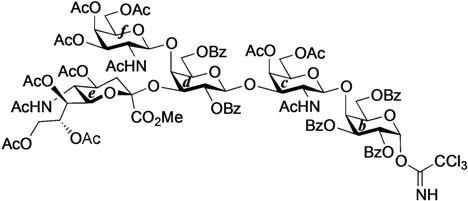


*2-Acetamido-3,4,6-tri-O-acetyl-2-deoxy-β-d-galactopyranosyl-(1→4)-{[methyl 5-acetamido-4,7,8,9-tetra-O-acetyl-3,5-dideoxy-d-glycero-α-d-galacto-2-nonulopyranosylonate]-(2→3)}-2,6-di-O-benzoyl-β-d-galactopyranosyl-(1→3)-2-acetamido-4,6-di-O-acetyl-2-deoxy-β-d-galactopyranosyl-(1→4)-2,3,6-tri-O-benzoyl-α-d-galactopyranosyl trichloroacetimidate* (**31**). To a solution of **30** (115 mg, 55.9 µmol) in acetonitrile/toluene/H_2_O (6:5:3, 1.1 mL) was added CAN (245 mg, 447 µmol) at 0 °C. After stirring for 2 h at 0 °C as the reaction was monitored by TLC (10:1 CHCl_3_–MeOH), the mixture was diluted with CHCl_3_. The solution was then washed with H_2_O, satd aq NaHCO_3_ and brine. The organic layer was subsequently dried over Na_2_SO_4_, and concentrated. The residue was roughly purified by silica gel column chromatography (30:1 CHCl_3_–MeOH). The product obtained was exposed to high vacuum for 20 h and then dissolved in CH_2_Cl_2_ (502 µL). CCl_3_CN (101 µL, 1.00 mmol) and DBU (9.0 µL, 60.2 µmol) were added to the mixture at 0 °C. After stirring for 30 min at rt as the reaction was monitored by TLC (15:1 CHCl_3_–MeOH), the reaction mixture was evaporated. The crude residue obtained was purified by silica gel column chromatography (40:1 CHCl_3_–MeOH) to give **31** (94 mg, 81%). [α]_D_ +14.0° (c 0.6, CHCl_3_); ^1^H-NMR (600 MHz, DMSO-*d*_6_) δ 9.65 (br s, 1H, C(=NH)), 8.01–7.37 (m, 26H, 5Ph, NHe), 7.21 (br s, 1H, NHc), 6.98 (d, 1H, *J*_2,NH_ = 8.2 Hz, NHf), 6.57 (br s, 1H, H-1b), 5.77 (br d, 1H, H-2b), 5.64 (near dd, 1H, H-3b), 5.31 (s, 1H, H-4c), 5.23 (m, 3H, H-3f, H-8e, H-4f), 5.10 (m, 2H, H-2d, H-7e), 4.89 (d, 1H, *J*_1,2_ = 7.6 Hz, H-1d), 4.86–4.81 (m, 3H, H-1f, H-1c, H-4e), 4.62 (m, 2H, H-4b, H-5b), 4.56 (br d, 1H, H-3d), 4.48 (m, 2H, H-6b, H-6d), 4.38 (near dd, 1H, H-6'b), 4.31 (br d, 1H, H-3c), 4.25 (near dd, 1H, H-6'd), 4.06 (m, 2H, H-6f, H-9e), 3.98–3.84 (m, 8H, H-6'f, H-9'e, H-6c, H-4d, H-5d, H-5f, H-2f, H-6'c), 3.81–3.69 (m, 6H, H-6e, OCH_3_, H-5e, H-5c), 3.62 (m, 1H, H-2c), 2.26 (near dd, 1H, H-3e*eq*), 2.09–1.66 (m, 37H, H-3e*ax*, 12Ac); ^13^C-NMR (150 MHz, CDCl_3_) δ 171.0, 170.8, 170.4, 170.4, 170.4, 170.3, 170.2, 169.8, 169.7, 166.1, 165.9, 165.3, 164.2, 160.4, 133.5, 133.3, 133.1, 133.0, 129.9, 129.8, 129.7, 129.6, 129.5, 128.7, 128.4, 128.4, 128.3, 128.2, 100.7, 100.5, 98.2, 98.0, 93.4, 90.8, 74.8, 73.5, 73.1, 71.9, 71.9, 71.4, 71.3, 70.9, 70.4, 70.1, 70.0, 69.5, 68.7, 67.2, 66.9, 66.4, 63.8, 63.5, 62.8, 62.2, 61.4, 55.3, 53.0, 51.2, 49.1, 36.1, 29.6, 23.3, 23.1, 22.9, 22.6, 21.0, 20.8, 20.8, 20.7, 20.6, 20.4, 20.3, 14.1. HRMS (ESI) *m*/*z*: found [M+Na]^+^ 2117.5111, C_95_H_105_Cl_3_N_4_O_43_ calcd for [M+Na]^+^ 2117.5110.


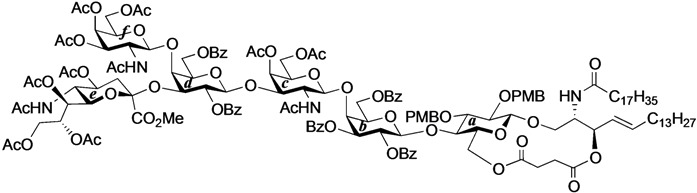


*4-O-{2-Acetamido-3,4,6-tri-O-acetyl-2-deoxy-β-d-galactopyranosyl-(1→4)-[(methyl 5-acetamido-4,7,8,9-tetra-O-acetyl-3,5-dideoxy-d-glycero-α-d-galacto-2-nonulopyranosylonate)-(2→3)]-2,6-di-O-benzoyl-β-d-galactopyranosyl-(1→3)-2-acetamido-4,6-di-O-acetyl-2-deoxy-β-d-galactopyranosyl-(1→4)-2,3,6-tri-O-benzoyl-β-d-galactopyranosyl-(1→4)}-2,3-di-O-p-methoxybenzyl-β-d-glucopyranosyl-(1'→1)-(2S,3R,4E)-2-octadecanamido-4-octadecene-1,3-diol-3,6'-succinate* (**32**). To a mixture of **31** (59 mg, 28.2 µmol) and **18β** (30 mg, 28.2 µmol) in CHCl_3_ (940 µL) was added 4 Å molecular sieves (100 mg) at rt. After stirring for 1 h, the mixture was cooled to 0 °C. TMSOTf (0.5 µL, 2.82 µmol) was then added to the mixture at 0 °C. After stirring for 1.5 h at rt as the reaction was monitored by TLC (1.5:1 acetone–*n*-hexane), the reaction was quenched by the addition of satd aq NaHCO_3_. The solution was diluted with CHCl_3_ and filtered through Celite. The filtrate was then washed with satd aq NaHCO_3_ and brine. The organic layer was subsequently dried over Na_2_SO_4_, and concentrated. The residue was purified by silica gel column chromatography (1:1 acetone–*n*-hexane) to give **32** (22 mg, 26%). [α]_D_ +7.8° (c 0.4, CHCl_3_); ^1^H-NMR (500 MHz, CDCl_3_) δ 8.08–6.80 (m, 33H, 7Ar), 6.77 (d, 1H, *J*_2,NH_ = 7.9 Hz, NHc), 5.73 (m, 2H, *J*_2,NH_ = 8.9 Hz, H-5*^Cer^*, NH*^Cer^*), 5.58–5.53 (m, 2H, H-3b, H-2d), 5.47–5.35 (m, 3H, NHf, H-3*^Cer^*, H-8e), 5.33 (d, 1H, *J*_3,4_ = 3.1 Hz, H-4c), 5.31–5.20 (m, 4H, H-4f, H-2b, H-4*^Cer^*, H-7e), 5.14 (d, 1H, *J*_5,NH_ = 9.9 Hz, NHe), 5.09 (d, 1H, *J*_1,2_ = 8.1 Hz, H-1f), 5.05 (d, 1H, *J*_1,2_ = 8.4 Hz, H-1c), 4.99 (m, 1H, H-4e), 4.96 (d, 1H, *J*_1,2_ = 7.8 Hz, H-1d), 4.84–4.73 (m, 3H, 2 ArC*H*_2_, H-3f), 4.71 (d, 1H, *J*_1,2_ = 7.7 Hz, H-1b), 4.66 (d, 1H, *J*_gem_ = 10.8 Hz, ArC*H*_2_), 4.61 (m, 2H, H-6b, H-6d), 4.52 (d, 1H, ArC*H*_2_), 4.47 (d, 1H, *J*_3,4_ = 1.6 Hz, H-4d), 4.33–4.19 (m, 5H, H-6'b, H-2*^Cer^*, H-1a, H-6f, H-6c), 4.14–4.03 (m, 5H, H-6'd, H-6'f, H-3c, H-9e, H-9'e), 4.00–3.87 (m, 6H, H-4b, H-6'c, H-6a, H-2c, H-3d, H-5e), 3.85–3.72 (m, 8H, H-6'a, OCH_3_, H-5b, H-6e, H-5d, H-1*^Cer^*), 3.66–3.58 (m, 3H, H-1'*^Cer^*, H-3a, H-4a), 3.54 (m, 1H, H-5a), 3.48 (m, 2H, H-5c, H-5f), 3.33 (t, 1H, *J*_1,2_ = *J*_2,3_ = 7.2 Hz, H-2a), 3.16 (m, 1H, H-2f), 2.58–2.45 (m, 4H, 2C(=O)CH_2_), 2.29 (dd, 1H, *J*_3eq,4_ = 4.8 Hz, *J*_gem_ = 13.4 Hz, H-3e*eq*), 2.18–1.55 (m, 43H, C(=O)CH_2_CH_2_*^Cer^*, H-3e*ax*, H-6*^Cer^*, H-6'*^Cer^*, 12Ac), 1.26 (m, 50H, 25-CH_2_-), 0.88 (m, 6H, 2-CH_3_*^Cer^*); ^13^C-NMR (125 MHz, CDCl_3_) δ 172.8, 171.8, 171.2, 171.0, 170.6, 170.5, 170.4, 170.4, 170.2, 169.9, 169.8, 168.1, 166.2, 166.0, 165.6, 164.3, 159.3, 159.0, 137.1, 133.6, 133.3, 133.1, 130.5, 130.0, 130.0, 129.8, 129.8, 129.7, 129.6, 129.6, 129.6, 129.0, 128.8, 128.5, 128.4, 128.4, 128.3, 128.2, 125.6, 124.3, 120.2, 113.8, 113.7, 101.8, 100.9, 100.6, 98.3, 97.8, 81.8, 80.7, 78.5, 77.6, 75.0, 73.9, 73.7, 73.5, 73.3, 72.7, 72.2, 72.0, 71.2, 70.6, 70.4, 70.4, 70.1, 70.0, 69.5, 68.7, 67.3, 66.9, 66.5, 63.5, 62.8, 62.7, 62.2, 61.4, 55.3, 55.2, 55.1, 53.8, 53.1, 51.9, 49.2, 43.0, 38.7, 36.6, 36.2, 32.3, 31.9, 31.7, 30.3, 29.7, 29.7, 29.7, 29.6, 29.6, 29.5, 29.4, 29.3, 29.3, 29.1, 29.0, 28.9, 28.8, 25.9, 25.6, 23.9, 23.8, 23.4, 23.1, 23.0, 22.9, 22.8, 22.7, 22.6, 21.1, 20.8, 20.8, 20.8, 20.6, 20.4, 20.4, 14.1, 14.1, 14.0, 11.0, 10.9. HRMS (ESI) *m*/*z*: found [1/2M+Na]^+^ 1514.6482, C_155_H_202_N_4_O_54_ calcd for [1/2M+Na]^+^ 1514.6484.


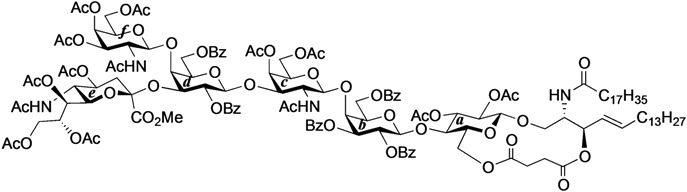


*4-O-{2-Acetamido-3,4,6-tri-O-acetyl-2-deoxy-β-d-galactopyranosyl-(1→4)-[(methyl 5-acetamido-4,7,8,9-tetra-O-acetyl-3,5-dideoxy-d-glycero-α-d-galacto-2-nonulopyranosylonate)-(2→3)]-2,6-di-O-benzoyl-β-d-galactopyranosyl-(1→3)-2-acetamido-4,6-di-O-acetyl-2-deoxy-β-d-galactopyranosyl-(1→4)-2,3,6-tri-O-benzoyl-β-d-galactopyranosyl-(1→4)}-2,3-di-O-acetyl-β-d-glucopyranosyl-(1'→1)-(2S,3R,4E)-2-octadecanamido-4-octadecene-1,3-diol-3,6'-succinate* (**33**). To a mixture of **31** (53 mg, 25.3 µmol) and **1** (23 mg, 25.3 µmol) in CHCl_3_ (843 µL) was added 4 Å molecular sieves (120 mg) at rt. After stirring for 1 h, the mixture was cooled to 0 °C. TMSOTf (0.5 µL, 2.53 µmol) was then added to the mixture at 0 °C. After stirring for 2.5 h at rt as the reaction was monitored by TLC (4:3 acetone–*n*-hexane), the reaction was quenched by the addition of satd aq NaHCO_3_. The solution was diluted with CHCl_3_ and filtered through Celite. The filtrate was then washed with satd aq NaHCO_3_ and brine. The organic layer was subsequently dried over Na_2_SO_4_, and concentrated. The residue was purified by silica gel column chromatography (1:1 acetone–*n*-hexane) to give **33** (22 mg, 31%). The yields of **33** based on the use of 2.0 eq. and 3.0 eq. of **1** were 48% and 60%, respectively. [α]_D_ +8.0° (c 0.5, CHCl_3_); ^1^H-NMR (600 MHz, CDCl_3_) δ 8.11–7.31 (m, 25H, 5Ph), 6.31 (d, 1H, *J*_2,NH_ = 8.2 Hz, NHc), 5.98 (d, 1H, *J*_2,NH_ = 6.2 Hz, NHf), 5.77 (m, 1H, H-5*^Cer^*), 5.59 (d, 1H, *J*_2,NH_ = 9.6 Hz, NH*^Cer^*), 5.52 (m, 2H, H-3b, H-2d), 5.38 (m, 1H, H-8e), 5.34–5.29 (m, 4H, H-4c, H-4f, H-2b, H-3*^Cer^*), 5.26–5.21 (m, 2H, H-4*^Cer^*, H-7e), 5.18–5.14 (m, 2H, H-3a, H-1f), 5.10 (d, 1H, *J*_5,NH_ = 9.7 Hz, NHe), 5.04 (d, 1H, *J*_1,2_ = 8.3 Hz, H-1c), 5.00 (m, 1H, H-4e), 4.88 (dd, 1H, *J*_3,4_ = 3.4 Hz, *J*_2,3_ = 11.0 Hz, H-3f), 4.83 (t, 1H, *J*_1,2_ = *J*_2,3_ = 7.2 Hz, H-2a), 4.75 (d, 1H, *J*_1,2_ = 7.6 Hz, H-1b), 4.69 (d, 1H, *J*_1,2_ = 7.6 Hz, H-1d), 4.62 (m, 2H, H-6b, H-6d), 4.52 (d, 1H, *J*_3,4_ = 2.8 Hz, H-4d), 4.34 (m, 3H, H-1a, H-6'b, H-6'd), 4.25 (m, 2H, H-2*^Cer^*, H-6f), 4.19 (near dd, 1H, H-6c), 4.20–4.05 (m, 4H, H-6´f, H-3c, H-6´c, H-9e), 4.02–3.88 (m, 6H, H-9'e, H-4b, H-6a, H-2c, H-3d, H-5e), 3.85–3.75 (m, 8H, H-1*^Cer^*, OCH_3_, H-1'*^Cer^*, H-5b, H-5d, H-6e), 3.70 (m, 2H, H-4a, H-6'a), 3.60 (m, 2H, H-5c, H-5f), 3.50 (near t, 1H, H-5a), 3.12 (m, 1H, H-2f), 2.59–2.40 (m, 4H, 2C(=O)CH_2_), 2.29 (dd, 1H, *J*_gem_ = 13.0 Hz, *J*_3eq,4_ = 4.8 Hz, H-3e*eq*), 2.19–1.55 (m, 49H, C(=O)CH_2_CH_2_*^Cer^*, H-3e*ax*, H-6*^Cer^*, H-6'*^Cer^*, 14Ac), 1.25 (m, 50H, 25-CH_2_-), 0.88 (m, 6H, 2-CH_3_*^Cer^*); ^13^C-NMR (150 MHz, CDCl_3_) δ 172.7, 171.3, 171.2, 171.1, 170.5, 170.4, 170.3, 170.3, 169.9, 169.7, 169.3, 168.1, 166.1, 166.0, 165.9, 164.9, 164.3, 133.6, 133.3, 133.2, 130.4, 130.1, 130.0, 129.8, 129.7, 129.5, 129.4, 129.0, 128.8, 128.7, 128.6, 128.5, 128.4, 128.2, 124.7, 101.0, 100.7, 100.4, 99.2, 98.3, 97.5, 76.5, 75.2, 74.0, 73.6, 73.3, 72.4, 72.1, 72.0, 72.0, 71.7, 71.3, 70.5, 70.4, 70.0, 69.6, 68.7, 67.3, 67.0, 66.5, 63.5, 63.0, 62.8, 62.2, 61.4, 53.1, 51.8, 50.0, 49.3, 36.8, 36.1, 32.3, 31.9, 29.7, 29.7, 29.5, 29.5, 29.4, 29.3, 29.3, 28.8, 25.6, 23.4, 23.2, 23.2, 23.1, 22.9, 22.8, 22.7, 22.6, 22.2, 22.0, 21.8, 21.1, 20.9, 20.8, 20.7, 20.6, 20.5, 20.4, 14.1. HRMS (ESI) *m*/*z*: found [1/2M+Na]^+^ 1436.6014, C_143_H_190_N_4_O_54_ calcd for [1/2M+Na]^+^ 1436.6015.


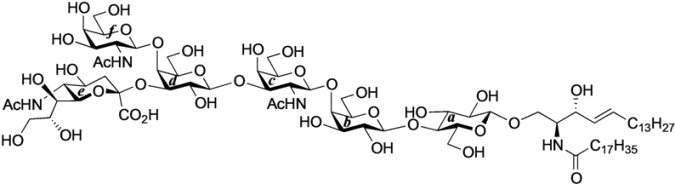


*GalNAc-GM1b: 2-acetamido-2-deoxy-β-d-galactopyranosyl-(1→4)-[5-acetamido-3,5-dideoxy-d-glycero-α-d-galacto-2-nonulopyranosylonic acid-(2→3)]-β-d-galactopyranosyl-(1→3)-2-acetamido-2-deoxy-β-d-galactopyranosyl-(1→4)-β-d-galactopyranosyl-(1→4)-β-d-glucopyranosyl-(1'→1)-(2S,3R,4E)-2-octadecanamido-4-octadecene-1,3-diol*. To a solution of **33** (15.0 mg, 5.31 µmol) in MeOH/THF (1:1, 532 µL) was added NaOMe (28% solution in MeOH, 102 µg, 0.531 µmol) at 0 °C. After stirring for 6 d at rt as the reaction was monitored by TLC (5:4:1 CHCl_3_–MeOH–10 mM aq ZnCl_2_), water (10 µL) was added to the mixture. After stirring for 8 d at rt, the reaction was neutralized with Dowex (H^+^) resin. The resin was filtered through cotton and the filtrate was then evaporated. The residue was purified by gel filtration column chromatography (LH-20) using CHCl_3_–MeOH as eluent followed by silica gel column chromatography (5:4:0.5 CHCl_3_–MeOH–H_2_O) to give the target **GalNAc-GM1b** (8.2 mg, 88%). [α]_D_ +12.5° (c 0.2, 1:1 CHCl_3_–MeOH); ^1^H-NMR (600 MHz, 1:1 CDCl_3_–CD_3_OD) δ 5.70 (m, 1H, H-5*^Cer^*), 5.45 (dd, 1H, *J*_3,4_ = 7.6 Hz, *J*_4,5_ = 15.1 Hz, H-4*^Cer^*), 2.73 (br d, 1H, H-3e*eq*), 2.18 (m, 2H, C(=O)CH_2_), 2.05–2.01 (m, 11H, 3Ac, H-6*^Cer^*, H-6'*^Cer^*), 1.85 (br t, 1H, H-3e*ax*), 1.59 (m, 2H, C(=O)CH_2_C*H*_2_), 1.37–1.19 (m, 50H, 25-CH_2_-), 0.89 (m, 6H, 2-CH_3_); ^13^C-NMR (150 MHz, 1:1 CDCl_3_–CD_3_OD) δ 174.8, 174.6, 173.7, 173.4, 134.4, 129.7, 129.5, 128.0, 104.4, 103.8, 103.1, 102.0, 79.0, 76.2, 75.2, 75.0, 74.7, 74.5, 73.8, 73.6, 73.5, 72.1, 71.9, 71.3, 69.6, 69.5, 68.7, 68.6, 68.2, 64.6, 62.0, 61.5, 60.4, 60.2, 53.3, 53.1, 52.6, 51.8, 47.7, 36.4, 32.4, 32.0, 29.7, 29.6, 29.6, 29.6, 29.4, 29.3, 26.1, 22.7, 22.7, 22.0, 13.8. HRMS (ESI) *m*/*z*: found [M−H]^−^ 1747.9487, C_81_H_144_N_4_O_36_ calcd for [M−H]^−^ 1747.9488.

## 4. Conclusions

In this study, we investigated the development of a GlcCer cassette acceptor that was both readily accessible and highly reactive. We designed and prepared a novel cassette acceptor bearing electron-donating PMB groups at C2 and C3 of the glucose residue. Various types of linkers and their effect on the stereoselectivity of intramolecular glycosylation were examined. Although varying the linker did not significantly increase β-selectivity, the use of a nitrile solvent gave predominantly the desired β-product. Considering the accessibility of the acceptor, we opted for the succinyl linker. In the experiment on coupling the cassette acceptor and oligosaccharide donor, we found that the use of PMB groups as protecting groups at C2 and C3 positions of the glucose residue did not enhance the reactivity as a GlcCer cassette acceptor. This interesting finding should provide useful information for the future design of glycosyl acceptors. Furthermore, we extended the generality of the GlcCer cassette approach by applying it to the efficient total synthesis of the ganglioside GalNAc-GM1b. Our laboratory is now conducting further studies to evaluate the scope and limitations of the GlcCer cassette approach.
